# Automated model discovery based on COVID-19 epidemiological data from Thuringia, Germany

**DOI:** 10.1371/journal.pone.0344409

**Published:** 2026-10-01

**Authors:** Morteza Babazadeh Shareh, Florian Kleiner, Michael Böhme, Corinna Hägele, Petra Dickmann, Rainer Heintzmann

**Affiliations:** 1 School of Computing and Communications, Lancaster University Leipzig, Leipzig, Saxony, Germany; 2 Leibniz Institute of Photonic Technology, Jena, Thuringia, Germany; 3 Institute of Inorganic and Analytical Chemistry, Friedrich Schiller University Jena, Jena, Thuringia, Germany; 4 Department of Anaesthesiology and Intensive Care, Jena University Hospital, Jena, Thuringia, Germany; 5 Leibniz Centre for Photonics in Infection Research (LPI), Jena, Thuringia, Germany; 6 Institute of Physical Chemistry and Abbe Center of Photonics, Friedrich Schiller University Jena, Jena, Thuringia, Germany; University of Missouri Kansas City, UNITED STATES OF AMERICA

## Abstract

The COVID-19 pandemic presented severe challenges in understanding and predicting the spread of infectious diseases, necessitating innovative approaches beyond traditional epidemiological models. This study introduces an advanced method for automated model discovery using the Sparse Identification of Nonlinear Dynamics (SINDy) algorithm, leveraging a dataset from the COVID-19 outbreak in Thuringia, Germany, encompassing more than 400,000 patient records and vaccination data. We develop a flexible, data-driven model capturing pandemic dynamics, incorporating external factors and interventions into the mathematical framework. The fixed coefficient values globally determined by SINDy were not accurate for local modelling of the data, a limitation of prior SINDy-based epidemiological applications that our framework directly addresses. We therefore refined our technique based on the differential equations as found by SINDy, by investigating three modifications that account for recent local data, each offering different strengths for short-term prediction, scenario analysis, and capturing nonlinear effects, achieving an R² of 0.87 at a one-week horizon. In a first approach, we re-optimized the coefficient values using seven days of past data, without changing the globally determined differential equation. In a second approach, we allowed a temporal dependence of the coefficient values, fitted using all previous data, in combination with regularization. As a last method, we kept the coefficients fixed to the original values but augmented the differential equation with a small neural network, locally optimized to the data of the past week. Our results link vaccination and public health measures to the pandemic's trajectory. The proposed model allows simulation of intervention scenarios, such as vaccination strategies and public health interventions. While the current study is based on retrospective data from a single region, the framework could serve as a basis for exploring responses to future outbreaks, subject to further validation.

## Introduction

The global outbreak of COVID-19 has underscored the critical importance of timely and accurate modelling in understanding and combating infectious diseases. Traditional mathematical models, such as the Susceptible-Infected-Recovered (SIR) model, have provided foundational insights into disease dynamics [1–3]. However, the complexity and unprecedented nature of the COVID-19 pandemic have revealed limitations in these classic approaches, particularly in their adaptability to rapidly changing data and their capacity to capture the complex interactions within and between populations [4]. To address these challenges, we use the Sparse Identification of Nonlinear Dynamical Systems (SINDy) algorithm [5] for automated model discovery based on COVID-19 data. By automated model discovery, we mean the process of extracting governing mathematical equations directly from measured data, without assuming a predefined equation structure — though, as with any SINDy application, this still requires specifying a library of candidate terms and a sparsity threshold. The SINDy algorithm analyses the data and determines which mathematical terms of a differential equation are best suited to explain the measured dynamics of the system. This algorithm starts with a matrix of the potential terms (called a basis) contributing to the desired differential equation model. By enforcing sparsity, it then determines a minimal set of required terms along with their coefficients. In this way, SINDy can extract the best differential equation approximating the system consistent with the measured data. Leveraging the power of this algorithm, we aim to transcend the constraints of conventional models, offering a more flexible and data-driven pathway to deciphering the complex dynamics of the disease spread. Despite these strengths, SINDy has recognised limitations: its results are sensitive to noise in the estimated derivatives, depend on the choice of candidate function library and polynomial order, and identifiability of the underlying equations is not guaranteed when multiple candidate structures fit the data comparably well [5]. We address several of these challenges through the pre-processing and validation steps described in Methods. In the rest of the section, we review previous approaches to COVID-19 modelling.

The limitations of traditional compartmental models have prompted researchers to explore enhancements by incorporating artificial intelligence elements. For example, in one COVID-19 modelling project, Alqahtani explores an innovative extension of the traditional SIR compartmental model by integrating fractional derivatives [6]. This approach offers a nuanced understanding of the epidemic's dynamics by incorporating memory effects, providing a more detailed analysis of infection spread over time. Alqahtani's work demonstrates the potential of modifying classic models to enhance their descriptive power, stability, and numerical analysis capabilities. However, this approach still requires the compartmental structure to be specified in advance and cannot discover mechanisms outside the SIR framework. This work underscores the importance of evolving mathematical frameworks to deal with the complex characteristics of the COVID-19 pandemic, serving as a good example of using advanced tools, such as the SINDy algorithm, for automated model discovery. In another study, Vega et al. made a significant leap towards integrating machine learning algorithms with the SIR epidemiological model. Their development of the SIMLR framework incorporates machine learning to refine COVID-19 forecasts, offering a novel method that enhances prediction accuracy by learning from the data in real-time [7]. This hybrid approach improves predictive accuracy, but the learned component does not yield an interpretable governing equation. This hybrid approach illustrates the potential of machine learning to augment traditional models and lays the groundwork for further explorations into adaptive, data-driven modelling techniques. Similarly, Kong et al. introduce a hybrid modelling strategy that combines the predictive strengths of epidemic differential equations with the adaptive learning capabilities of recurrent neural networks (RNNs). This method significantly advances the accuracy of COVID-19 prevalence forecasts and shows the potential of hybrid models in navigating the complexities of pandemic dynamics [8]. By leveraging the structured insights of epidemic models with the flexible, data-driven nature of recurrent neural networks (RNNs), their work paves the way for more resilient forecasting tools. However, the RNN component remains a black box, offering limited mechanistic insight into the source of the improved accuracy. In a similar direction, Cheng et al. combined a SEIRV compartmental model with deep neural networks to forecast COVID-19 spread, showing that hybrid architectures can improve prediction accuracy over purely mechanistic models [9]. Similarly, the deep-learning component here is not interpretable, limiting mechanistic insight despite the accuracy gain. Similar modelling challenges have been identified in other infectious diseases, such as Plasmodium vivax transmission, where a recent scoping review highlighted the need for more flexible and data-driven mathematical frameworks [10].

Recent research aims to shed light on the importance of the interventions and external factors in the dynamics of the pandemic. In one study, Dehning et al. contributed significantly to the understanding of the temporal dynamics of COVID-19 through the lens of change-point analysis. By accurately analysing the effects of public health interventions on the pandemic's trajectory, this research offers insights into how timely and targeted measures can alter the course of the disease's spread. The study provides a quantitative framework for evaluating intervention strategies and highlights the critical need for adaptable and data-informed models in public health planning [11]. Another study has comprehensively explored the role of epidemiological models in analysing the complex dynamics of the COVID-19 pandemic [12]. This research uses modelling to monitor and predict the outbreak's trajectory, evaluating the efficacy of public health interventions, and informing policy decision makers. By synthesising data across various scales and contexts, the study highlights the essential value of epidemiological models in offering insights critical to shaping a coordinated and effective response to the pandemic. A pivotal study [13] explores the global impact of vaccine distribution strategies using an advanced version of the SIR model. By analysing data from 152 countries in 2021, the potential benefits of equitable vaccine sharing in reducing the global burden of COVID-19 were described. Their findings underscore the importance of prioritising need over wealth in vaccine distribution to mitigate the spread of the virus and advocate for equitable solutions in the pandemic response. The studies above illustrate the importance of considering external factors in pandemic modelling. The ability of the SINDy algorithm to accept control signals as external factors helps us incorporate such external factors [5].

Given the limitations of the current mathematical pandemic models, some researchers are trying to develop methods based on purely data-driven approaches. For example, in [14], the authors present a novel approach to forecast virus outbreaks by leveraging social media data and neural ordinary differential equations (NODEs). By integrating real-time information from social media platforms into their modelling framework, the researchers demonstrate the potential for improved epidemic forecasting accuracy. Their use of NODEs allows for dynamic modelling of complex, nonlinear interactions, enabling more precise predictions of outbreak trajectories. This innovative methodology highlights the valuable insights that can be gathered from unconventional data sources and underscores the importance of flexible modelling approaches in addressing dynamic public health challenges. However, their method relies heavily on recent data (two months) and does not yield a globally valid model of the underlying epidemic dynamics. Gao et al. introduce an evidence-driven spatiotemporal prediction model leveraging Ising dynamics [15] for COVID-19 forecasting. By integrating diverse data sources and employing Ising dynamics, a statistical physics approach, their model provides insightful predictions of COVID-19 hospitalisations at both spatial and temporal scales. This methodology accounts for complex interactions between various epidemiological factors and geographical regions, offering a nuanced understanding of disease dynamics. The study's emphasis on evidence-driven predictions highlights the importance of data-driven approaches for effective public health interventions. Sandie et al. present an analysis of the observed versus estimated trends in COVID-19 case numbers in Cameroon [16]. They use a statistical technique, Multilevel Regression with Poststratification (MRP), and then apply Seasonal Autoregressive Integrated Moving Average (SARIMA) analysis to provide valuable insights into the pandemic dynamics. By comparing actual case data with model predictions, they evaluate the forecasting accuracy of existing modelling frameworks. This research sheds light on the challenges and limitations inherent in pandemic modelling, particularly in resource-constrained settings. From these studies we learn that refined modelling techniques and incorporating local data to improve the accuracy of disease forecasts are essential.

Taken together, these studies suggest that standard SIR-family models may be insufficient to capture the full complexity of the COVID-19 pandemic, calling for improvements. Despite making valuable contributions, these methods still rely on predefined model structures (such as SIR and its extensions) or use machine learning as an add-on to improve predictions without changing the underlying equations. In contrast, our approach does not assume a specific equation structure, although, like other SINDy applications, it still requires specifying the library of candidate terms from which that structure is selected. Instead, we let the SINDy algorithm determine the governing equations directly from the data. Furthermore, while purely data-driven methods such as neural ordinary differential equations can achieve good predictive accuracy, the resulting models are typically black boxes that offer limited insight into the underlying dynamics. SINDy bridges this gap by producing explicit mathematical equations that are both data-driven and interpretable. Therefore, we aim to automatically extract mathematical models capable of capturing the complex features of the pandemic directly from the data using SINDy. We expect that this yields mathematical models that inherently represent the pandemic system, eliminating the need for auxiliary methods to enhance model performance. We apply a data-driven approach to a local dataset aiming to extract the mathematical model automatically. Using a data-driven approach avoids making prior assumptions about the role of different variables in the system before constructing the model. We instead let the model discovery algorithm analyse the (Thuringian) dataset to construct a suitable pandemic model, noting that the discovered equations are specific to this regional context.

In this work, we use pre-processing methods to reduce the noise and make the data more suitable for modelling. Moreover, we apply optimisation methods to make the final model more accurate and capable of capturing temporal external factors, connecting the discovered equations and their coefficients to their epidemiological meaning and public health implications. The rest of the paper is organised as follows: First, we explain the method, then some results from the proposed method, and finally, in the conclusion section, we discuss our work.

## Materials and methods

Fig 1 provides an overview of the methodological framework employed in this study, which begins with a pre-processing stage, followed by mathematical modelling and model optimisation. In the following, we first describe the data; then present a high-level description of the method, and finally detail each step of the pipeline.

**Fig 1:**
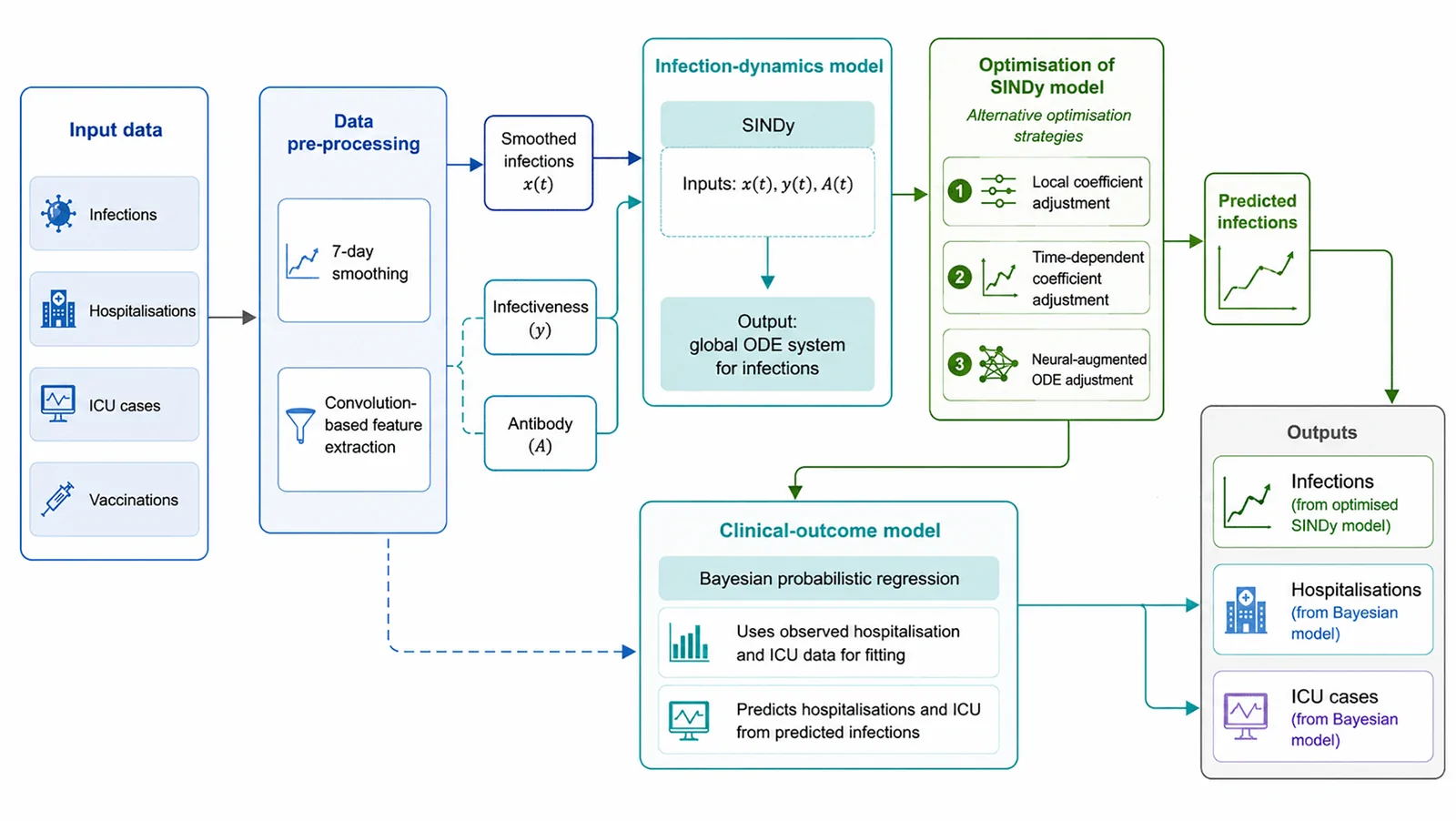
Overview of the proposed model. Raw epidemiological and vaccination data are pre-processed to derive infectiveness and antibody features, followed by SINDy-based modelling of infection dynamics, Bayesian prediction of hospitalisation and ICU cases, and three alternative strategies for adapting the SINDy model to recent or time-varying dynamics.

### Data

The primary time-series dataset was derived from COVID-19 patient records gathered by the local health authorities of the Free State of Thuringia according to the German Infection Protection Act, combined by the Thuringian State Authority for Consumer Protection (TLV), and curated by the Pandemics Control Group, starting on the 3^rd^ of March 2020 (the first SARS-CoV-2 case in Thuringia, Germany) and ending on the 7^th^ of February 2022. The data used in this study were collected by Thuringia's local health offices as part of their legal duty to report infections under the German Infection Protection Act, not for research purposes, and were fully anonymised before the authors received them. Because this is a secondary analysis of anonymised public health data rather than a study involving direct contact with patients, no separate ethics committee approval or informed consent was required. Access to the anonymised data was authorised by the Thuringian State Authority for Consumer Protection (TLV). Thuringia is a federal state in central Germany with about 2.1 million inhabitants and a relatively high average age of around 47 years, according to the Thuringian State Office for Statistics (TLS, 2022).

The raw dataset comprised roughly 400,000 anonymous records from patients in various districts. Every record contained detailed information such as infection date, age, gender, vaccination, and hospitalisation status. This information was processed to build a comprehensive time-series dataset of infection, hospitalisation and admission to the intensive care unit (ICU) stratified by age group. No individual records were excluded during processing. Reporting artefacts and day-of-week fluctuations in the resulting daily counts were addressed using the 7-day moving-average smoothing, rather than through record-level exclusion or imputation.

This primary dataset covers more than two years of the pandemic records. However, the quality of these data must be considered with caution. Actual infection numbers were likely underestimated, particularly during peak infection periods when registration offices were overwhelmed, and in later stages of the pandemic when many individuals chose not to report their infections. The effect of underreporting is especially noticeable around Christmas and New Year, where a sharp decline in reported cases can be observed.

Another set of information we used in the model is the overall vaccination records in Thuringia. These data start on the 27^th^ of December 2020 (first jab in Thuringia) and end on the 30^th^ of June 2022. The raw vaccination data, gathered by the health authorities of the Free State of Thuringia and curated by the Pandemics Control Group, recorded all vaccination centre activities in the state. Each centre recorded on a daily basis how many people, at which age, got vaccinated and whether it was their first, second, or third jab. After processing these data, we built a comprehensive time-series dataset describing all vaccinations in the state.

### Method description

We aim to predict the number of infections, hospitalisations, and ICU cases for a specific time range. The main approach of the proposed method is to develop a model that captures the dynamic patterns of the pandemic, including its peaks, declines, and fluctuations over time. As Fig 1 shows, the proposed method has three steps: we first pre-process the data to reduce the noise in the dataset and extract two new features (Infectiveness and Antibody) using the measured information. The reported case numbers showed a clear dependence on the day of the week of reporting, which we corrected using a sliding-window weekly average. The robustness of this choice to shorter and longer averaging windows was subsequently assessed and is reported in the Results (Table 6). This single weekly averaging step also mitigated delays in reporting during holiday periods, particularly around Christmas and Easter. In the pre-processing, we also convolved the data to extract two new features (Infectiveness and Antibody). The second step was to automatically discover a mathematical model representing the dynamics of COVID-19 infections in Thuringia. This mathematical model has two parts. The first part consists of a number of ordinary differential equations extracted from the time-series dataset. These ODEs modelled only the number of infections. We used the SINDy algorithm to discover these equations. The second part of the mathematical model aims to predict the number of hospitalisation and ICU cases. Since we observed a linear correlation between hospitalisations (and ICU cases) and infections, Bayesian statistics were used to implement a probabilistic regression on the data. The third and final step of our method is to optimise the coefficients in the differential equations based on more recent time stretches of the data (one week), to allow better predictions. The extracted ODEs using SINDy provided an overall model of the pandemic's non-linear behaviour. Yet, when we want to make a projection from a particular starting date, we expect that external temporary confounding factors (like a period of public health intervention) may have significant influence on the dynamics. Therefore, when projecting from a certain date into the future, we first optimised the coefficients of the ODEs based on one week of data points before that date. In this way, such recent influences on the pandemic were accounted for. In the following section, each step is discussed in detail. Table 1 lists the symbols used in the equations throughout this section.

**Table 1:** Definitions of variables, parameters, and functions used in the Methods section.

Symbol	Meaning
t	Time step
n	Total number of time steps in the dataset
x	Full time series of infections (smoothed)
y	Full time series of infectiveness
v	Full time series of vaccinations (smoothed)
h	Full time series of hospitalisations (smoothed)
u	Full time series of ICU cases (smoothed)
A	Full time series of antibody
x(t)	Number of infections at time t (smoothed)
y(t)	Infectiveness at time t
v(t)	Number of vaccinations at time t (smoothed)
h(t)	Number of hospitalisations at time t (smoothed)
u(t)	Number of ICU cases at time t (smoothed)
A(t)	Antibody at time t
$\tilde{d}\left(t\right)$	Any noisy data item at time t
d(t)	Any smoothed data item at time t
k	Number of days an individual is infectious or retains antibodies
beta(α,β)	Beta distribution with shape parameters α and β
pdf	Probability density function evaluated over the Beta distribution
$\varphi_{i}$	Value of the Beta PDF at position $\frac{\left(i-\text{1}\right)}{\left(k-\text{1}\right)}$
φ	Kernel array of length k with values sampled from the Beta pdf
$\varphi^{\left(inf\right)}$	Convolution kernel for infections to calculate infectiveness
$\varphi^{\left(vecc\right)}$	Convolution kernel for vaccinations to calculate antibody
⨂	Convolution operator
X	Matrix of state variables (x(t),y(t))
$\dot{X}$	Matrix of time derivatives of state variables over n time steps (size n×m)
θ(X)	Library of b candidate basis functions evaluated from X (size n×b)
Ξ	Coefficient matrix containing the sparse weights for each equation (size b×m)
m	Number of modelled state variables (here x(t),y(t))
b	Number of candidate basis functions in Θ(X)
$\mu_{t}^{\left(h\right)},\mu_{t}^{\left(u\right)}$	Expected value of hospitalisation and ICU cases at time t
$\alpha_{h},\alpha_{u},\beta_{h},\beta_{u}$	Intercept and slope parameters; assigned positive half normal $N^{+(\text{0},\cdot )}$
$\sigma_{h},\sigma_{u}$	Observation noise; assigned uniform priors
D	Prediction date
K	Number of time steps used for local optimisation
W	Current coefficient vector
$W_{SINDy}$	Original coefficients learned by the SINDy
$c_{t}$	Coefficient vector at time t
c	Full set of time-dependent coefficient vectors $\left\{c_{\text{1}},c_{\text{2}},\ldots c_{n}\right\}$
$\lambda_{w}$	Regularisation parameter for deviation from $W_{SINDy}$
$\lambda_{tv}$	Regularisation parameter for total variation across time
$f\left(t,W\right),f\left(t,c_{t}\right)$	Model prediction at time t with the coefficients Wand $c_{t}$
${\left\|\cdot \right\|}_{1},{\left\|\cdot \right\|}_{2}$	L1 and L2 norms
$f_{1},f_{2}$	Known symbolic part of the ODE from SINDy
$g_{1},g_{2}$	Neural network approximator for unknown dynamics
$\theta_{1},\theta_{2}$	Trainable parameters of the neural network

### Step 1: Data pre-processing

The dataset must first be pre-processed to become suitable for the SINDy algorithm. Two distinct issues in the dataset need to be addressed before the next step. One of the critical problems in the raw dataset is biases in reporting infections, hospitalisations, ICU cases and vaccinations. Since many healthcare offices were closed on weekends, the weekend cases were often reported in the first days of the following week. This reporting bias caused strong fluctuations in the data. To overcome this problem, we smoothed the data. Since the SINDy algorithm relies on numerical differentiation of the time-series data, it is sensitive to noise and reporting artefacts. Therefore, this averaging step helps to stabilise the derivatives and leads to more reliable model discovery. We acknowledge that averaging the measured data can lead to biased derivatives. However, the discovered model incorporates these biases, which are expected to be small since the averaging window is significantly smaller than the dominant time scales of the pandemic dynamics. A more detailed validation, varying the window size, can be found in the results section. According to the nature of the reporting bias, which varied with the day of the week, a weekly averaging box-filter of uniform weight (Eqn 1) was applied to the raw data, where $\tilde{d}(t)$ is any noisy data (infections, hospitalisations, ICU cases and vaccinations) reported at time *t*. The output *d(t)* is the smoothed version of this data. Here, *n* is the total number of data points in the time series data. The value of six reflects the seven-day averaging window. Throughout this paper all variables (e.g., *x(t), u(t)*) refer to smoothed data series unless explicitly marked with a tilde (e.g., $\tilde{x}(t)$) to indicate raw data.


$$\begin{array}{c} d(t)=\frac{\text{1}}{t-\text{max}(\text{1},t-\text{6})+\text{1}}\sum_{i=\text{max}(\text{1},t-\text{6})}^{t}\tilde{d}(i)\;where\;t=\text{1}\;to\;n\; \end{array}$$ (1)

Since SINDy discovers only ordinary differential equations (ODEs) without hidden states, it cannot directly represent delays, such as the time between infection and the onset of infectiveness. To account for such delays, we incorporated them during the pre-processing step by convolving the measured time series (infections and vaccinations) with appropriate kernel functions. For each kernel, we chose a Beta distribution, which is defined by two shape parameters, *α* and *β*, and allows us to generate flexible bell-shaped curves [17]. Equation (2) defines a *β* -distribution based kernel function denoted as *ϕ*.


$$\begin{array}{c} {\varphi =\left[\varphi_{\text{1}},\varphi_{\text{2}},\ldots ,\varphi_{k}\right]\in R^{k},\text{ where}\varphi }_{i}=\text{pdf}\left(\text{beta}(\alpha ,\beta ),\frac{i-\text{1}}{k-\text{1}}\right),i=\text{1}..k\; \end{array}$$ (2)

The vector *ϕ* consists of *k* values sampled from the Beta distribution's probability density function (PDF), where *k* is the number of days during which an infected person is considered infectious, or a vaccinated person retains antibodies. The kernel *ϕ* captures how infectiveness or antibody levels evolve over time after the respective event. Each point *ϕ*_*i*_ corresponds to the value of the Beta PDF evaluated at the normalized position *(i-1)/(k-1)*, ensuring the kernel spans the full interval *[0,1]* and forms a smooth curve suitable for convolution.

Equations (3) and (4) show how the convolution is carried out on the data. *Φ*^*(inf)*^ is the kernel used to convolve the infections, and *ϕ*^*(vacc)*^ is for the vaccinations. *x* and *v* are the full smoothed time series of the infections and vaccinations data respectively, and the outputs *y* and *A* we call “infectiveness” and “antibody”.


$$\begin{array}{c} y(t)=x⊗\varphi^{\left(inf\right)} \end{array}$$ (3)

$$\begin{array}{c} A(t)=v⊗\varphi^{\left(\text{vacc}\right)} \end{array}$$ (4)

### Step 2: Mathematical modelling

The output of the previous step is a smoothed version of the dataset, enriched with additional informative features (the infectiveness and antibody). In this step, we construct a mathematical model based on the pre-processed time-series data. Our goal is to predict infections, hospitalisations, and ICU cases. Modelling all three variables simultaneously was found to be too complex and potentially underdetermined, so we adopted a hierarchical approach. We first use the SINDy algorithm to identify the underlying differential equations governing the infection dynamics. In simple terms, SINDy works by constructing a large list of candidate mathematical terms (such as x, y, xy, x^2^, etc.) and then selecting only the few terms that are needed to describe the observed data. The result is a compact set of equations that captures the essential dynamics of the system. Based on the observed linear correlation between infections and subsequent hospitalisations (including ICU admissions), we then use the predicted infections to estimate the other variables.

We begin by presenting the SINDy-based modelling of infections, followed by the prediction of hospitalisations and ICU cases. Equation (5) presents the sparse regression framework used by SINDy to identify a compact system of differential equations that best captures the infection dynamics.


$$\begin{array}{c} \dot{X}=\theta (X)\cdot \Xi \; \end{array}$$ (5)

Equation (5) expresses the core structure of the SINDy algorithm as a sparse regression problem. The matrix $\dot{X}\in {\mathbb{R}}^{n\times m}$contains the numerically computed time-derivatives of the state variables (namely infections *x(t)* and infectiveness *y(t)*) evaluated over *n* time steps. The matrix $\theta (X)\in {\mathbb{R}}^{n\times b}$ is the library of the *b* candidate basis functions constructed from the state variables and the external input *A(t)*, which represents the antibody level. Although *A(t)* is not part of the state dynamics being directly modelled, it is included as a control signal influencing the system. The matrix $\Xi \in {\mathbb{R}}^{b\times m}$contains the sparse coefficients that determine the contribution of each basis function to the differential equations governing *x(t)* and *y(t)*. Here, *n* is the number of time points, *m* is the number of modelled variables, and *b* is the number of basis functions in the library.

To estimate *Ξ*, the SINDy algorithm solves a sparse regression problem using the Sequentially Thresholded Least Squares (STLSQ) method. This method includes a threshold parameter *λ* which controls the sparsity of the solution. A higher *λ* yields a sparser model with fewer active terms but may reduce accuracy, whereas a lower *λ* results in a more complex model that better fits the data. We solved the problem by using different values of *λ* and selected the Pareto-optimal solution as the final model. The specific parameter values used are reported in Table 2 (Results).

**Table 2:** The parameters of the SINDy.

Parameter	Value
**Type of the Problem**	**Continuous**
**Variables**	infections, infectiveness (x,y)
**Control Signal**	antibody (A)
λ	$\left[{\text{1}\text{0}}^{-\text{5}},{\text{1}\text{0}}^{-\text{1}}\right]$
**Basis**	Polynomial (quadratic)

After identifying a suitable model for infections via SINDy, the second part of the modelling process focuses on predicting the number of hospitalisations and ICU cases. Because a strong correlation between hospitalisations (and ICU cases) and infections is expected, we use a linear model based on probabilistic regression. Bayesian statistics are employed to account for uncertainty. Equation (6) shows how hospitalisations and ICU cases are estimated from the infection data:


$$\begin{array}{c} h(t)\;\sim \;N\left(\mu_{t}^{\left(h\right)},\sigma_{h}^{\text{2}}\right),\;\;\mu_{t}^{\left(h\right)}=\alpha_{h}+\beta_{h}\cdot x(t) \end{array}$$ (6)

$$\begin{array}{c} u(t)\;\sim \;N\left(\mu_{t}^{\left(u\right)},\sigma_{u}^{\text{2}}\right),\;\;\mu_{t}^{\left(u\right)}=\alpha_{u}+\beta_{u}\cdot x(t) \end{array}$$

Equation (6) defines a probabilistic linear regression model used to predict hospitalisations, *h(t)*, and ICU cases, *u(t)*, based on the number of infections, *x(t)*, at time *t* and *N(μ, σ²)* denotes the normal distribution with expectation *μ* and variance *σ²*. Each of the two observed outcomes is assumed to follow a normal distribution, centred at a linear mean function: *μ_t_^(h)^ = α_h_ + β_h_· x(t)* for hospitalisation, and *μ_t_^(u)^* = α_*u*_ + β_*u*_*· x(t)* for ICU cases. The parameters *α* and *β* represent the intercept and slope of each linear relationship and are assigned positive half-normal priors *N ⁺ ^(0,...)^* to enforce nonnegativity. The noise parameters *σ*_*h*_ and *σ*_*u*_, which capture the uncertainty in observations, are drawn from uniform distributions. This Bayesian regression framework enables uncertainty-aware prediction of hospitalisation and ICU trends based on infection counts. This model is applied across all time points *t = 1, 2, …, n*, allowing the regression parameters *α*, *β* and *σ* to be inferred from the full infections and hospitalisations (or ICU cases) time series. Posterior inference was performed using the No-U-Turn Sampler (NUTS), a Hamiltonian Monte Carlo method that adaptively tunes step size and trajectory length for efficient sampling. Convergence was assessed using the Gelman–Rubin statistic (R̂), with values close to 1 indicating agreement between chains.

### Step 3: Optimisation

The system of differential equations obtained via SINDy provides a global model of the pandemic dynamics, incorporating the antibody level as an external control input. However, the model coefficients may also be influenced by unobserved or time-varying external factors, such as policy changes or shifts in public behaviour. As a result, while the SINDy model captures the overall trends of the system, discrepancies between its predictions and the observed data can still occur.

To address these deviations, we allow the coefficients of the ODEs to adapt over time in response to such external influences, while keeping the structural form of the equations fixed. For this purpose, we applied three different optimisation strategies, each designed to fine-tune the model parameters and improve predictive performance under dynamically changing conditions. These strategies are referred to as:

1. Local coefficient adjustment,
2. Time-dependent coefficient adjustment, and
3. Neural-augmented ODE adjustment.

These three strategies were chosen because they complement each other and cover different levels of complexity. They all share one principle: the structural form of the differential equations discovered by SINDy is preserved, so the model remains interpretable. The local coefficient adjustment provides a simple and fast correction based on recent data. The time-dependent coefficient adjustment captures gradual changes caused by external factors and allows counterfactual scenario analysis. The neural-augmented ODE adjustment offers the most flexibility in accounting for complex nonlinear effects. Alternative approaches, such as Kalman filters or ensemble methods, were not used because they would either require hidden states or change the structure of the discovered equations.

We refer to the original, unoptimised coefficients obtained directly through SINDy as the global coefficients. The details of each optimisation strategy are presented in the following paragraphs. The corresponding hyperparameters for each strategy are reported in Tables 3 and 4 (Results).

**Table 3:** Optimisation process parameters for local and time-dependent coefficient adjustment.

Parameter	Value
**Loss Function**	Mean Squared Error (MSE)
**Differentiation**	Forward Differentiation
**ODE Solver**	Tsitouras 5^th^ Order Runge-Kutta (Tsit5)
**Iterations**	${\text{1}\text{0}}^{\text{3}}$
**Optimiser**	Broyden-Fletcher-Goldfarb-Shanno (BFGS)
**Penalty Function (Local Coefficient Adjustment)**	Squared Euclidean Distance
**Penalty Function (Time-dependent Coefficient Adjustment)**	Total Variation Regularisation (TVR)

**Table 4:** Optimisation parameters for the neural-augmented ODE adjustment, using UDE.

Parameter	Value
**Loss Function**	Mean Squared Error (MSE)
**NN Structure**	2x5x5x5x5x2
**Activation Function**	The Radial Basis Function (RBF)
**Iteration**	${\text{1}\text{0}}^{\text{3}}$
**ODE Solver**	Tsitouras 5th Order Runge-Kutta (Tsit5)
**Optimiser 1**	Adaptive Moment Estimation (ADAM)
**Learning Rate Optimiser 1**	0.001
**Optimiser 2**	Broyden-Fletcher-Goldfarb-Shanno (BFGS)

Equation (7) defines the local coefficient adjustment approach to optimise the coefficients *W* of the SINDy-based model for improved short-term prediction. The objective function minimises the squared error between the model output *f(t,W)* and the observed infection data *x(t)* over the most recent *K* time steps, ending at the prediction date *D*. A regularisation term, weighted by *λ*_*w*_, penalises deviations from the original SINDy coefficients *W*_*SINDy*_, ensuring that the locally optimised model remains close to the globally learned dynamics.


$$\begin{array}{c} \underset{w}{min}\left(\sum_{t=D-K+\text{1}}^{D}{\left(x(t)-f(t,W)\right)}^{\text{2}}+\lambda_{w}{\left\|W-W_{\text{SINDy}}\right\|}_{\text{2}}^{\text{2}}\right)\; \end{array}$$ (7)

Equation (8) shows the time-dependent coefficient adjustment approach, which allows the model’s coefficients *c*_*t*_ to change over time in response to external influences. The objective function minimises the squared error between the model output *f(t, c*_*t*_*)* and the observed infection data *x(t)* over all time steps up to the prediction date *D*. To ensure temporal smoothness and prevent overfitting, a total variation regularisation term is added. This term penalises abrupt changes between consecutive coefficient vectors *c*_*t*_ and *c*_*t-1*_, scaled by the regularisation parameter *λ*_*tv*_. In this approach, to make a projection from a specific date *t*, the corresponding coefficient vector *c*_*t*_ at that time is used in the model.


$$\begin{array}{c} \underset{{\{c}_{t}\}}{min}\;\left(\sum_{t=\text{2}}^{D}{\left(x(t)-f(t,c_{t})\right)}^{\text{2}}+\lambda_{tv}\sum_{t=\text{2}}^{D}{\left\|c_{t}-c_{t-\text{1}}\right\|}_{\text{1}}\right) \end{array}$$ (8)

Equation (9) defines the neural-augmented ODE adjustment approach, in which the original differential equations obtained by SINDy are extended with neural networks to capture additional time-dependent influences not explicitly modelled. The functions *f*_*1*_ and *f*_*2*_ represent the deterministic dynamics learned from the SINDy model, while the neural networks *g*_*1*_ and *g*_*2*_, parameterised by *θ*_*1*_ and *θ*_*2*_, are trained to approximate unknown external factors. All components are evaluated using the time-dependent variables *x(t)*, *y(t)* and *A(t)* where *x(t)* and *y(t)* are infections and infectiveness respectively and *A(t)* is the antibody level as a control input. The neural terms allow the model to adapt to dynamic effects without changing the original ODE. This approach is implemented using the Universal Differential Equations (UDE) framework, which enables the hybrid integration of mechanistic and data-driven components [18].


$$\begin{array}{c} \frac{dx(t)}{dt}=f_{1}\left(x(t),y(t),A(t)\right)+g_{1}\left(x(t),y(t),A(t);\theta_{1}\right) \end{array}$$ (9)

$$\begin{array}{c} \frac{dy(t)}{dt}=f_{2}\left(x(t),y(t),A(t)\right)+g_{2}\left(x(t),y(t),A(t);\theta_{2}\right) \end{array}$$

In summary, the local coefficient adjustment is fast and effective for short-term recalibration, especially in low-incidence periods, but it only uses a short window of past data. The time-dependent coefficient adjustment captures gradual changes over the full pandemic timeline and enables scenario analysis, but it requires optimisation over all previous time points. The neural-augmented ODE adjustment is the most flexible and handles complex nonlinear effects, but it is computationally more expensive and less interpretable than the other two. In terms of computational cost, the local coefficient adjustment is the cheapest, requiring only a small parameter-estimation problem over a fixed 7-day window; its training time and scalability are therefore stable, since the cost does not grow as more pandemic data accumulates over time. The time-dependent adjustment has moderate training time per fit, but its computational cost scales with the length of the historical time series, since each fit uses all previous data. The neural-augmented adjustment has the highest training time of the three, as it requires iterative gradient-based training of a neural network on each 7-day window rather than a small closed-form or low-dimensional fit; its scalability, however, is similar to the local adjustment, since it also operates on a fixed-size window regardless of the pandemic's overall duration.

### Computational implementation

All analyses were implemented in Julia (v1.11.5). Data handling used DataFrames.jl (v1.7.0 [19],) and CSV.jl. The SINDy model discovery (Step 2) used DataDrivenDiffEq.jl (v1.8.0 [25],) together with DataDrivenSparse.jl for the STLSQ sparse regression, and Distributions.jl [24] for constructing the Beta-distribution convolution kernels. Numerical integration of the ODE/SDE systems used DifferentialEquations.jl (v7.16.1 [26],); stochastic simulations used the Euler–Maruyama (EM) method from the same package's StochasticDiffEq.jl component. Coefficient optimisation (Step 3) used DiffEqParamEstim.jl (v2.2.0 [28],) for the local coefficient adjustment and Optim.jl [29] for the time-dependent coefficient adjustment; the neural-augmented ODE adjustment used Lux.jl [30] for the network architecture and Optimization.jl [31] for training. The Bayesian regression for hospitalisation and ICU prediction (Eq 6) used Turing.jl [27] with the NUTS sampler. Random seeds were fixed for the stochastic components of the pipeline to support reproducibility. The complete, version-pinned software environment and all analysis notebooks are available in the project repository (see Data Availability).

### Model evaluation

The four prediction approaches were assessed using walk-forward validation (WFV), in which the test window is moved forward through the dataset rather than randomly partitioned, preserving temporal ordering and better reflecting real-world forecasting conditions. The specific window length and step size are reported in Results. Overfitting was addressed differently across the three optimisation strategies. For the local and time-dependent coefficient adjustments, the regularisation terms in Equations 7 and 8 (weighted by λw and λtv respectively) constrain the optimised coefficients from deviating excessively from the SINDy baseline or from changing abruptly between time steps. For the neural-augmented ODE adjustment, the small network architecture and a fixed number of training iterations serve as the primary safeguards against overfitting to the short training window. Across all three strategies, generalisation was assessed out-of-sample via the walk-forward validation described above, rather than on the data used for fitting.

### Sensitivity analysis

To assess the robustness of the discovered model, three sensitivity analyses were performed. First, the sensitivity of the SINDy model structure to the smoothing window length was tested by repeating the full model discovery pipeline with shortened and prolonged averaging windows, checking whether the same set of active terms and coefficient signs were recovered. Second, the sensitivity to the convolution kernel parameters was assessed by varying the Beta distribution shape parameters of the infectiveness and antibody kernels around their baseline values and repeating the model discovery pipeline. Third, the sensitivity of the model's predicted dynamics to its coefficients was evaluated by perturbing each coefficient of the discovered differential equations and observing the resulting change in predicted trajectories. Full parameter ranges, evaluation criteria, and results for each analysis are reported in Results.

### Workflow summary

The pipeline can be summarised as follows: (1) raw case and vaccination data are smoothed via a 7-day moving average; (2) two convolution kernels (Beta(2.5, 4.5), k = 21 days for infectiveness; Beta(3, 4), k = 35 days for antibody) derive the infectiveness and antibody features; (3) SINDy performs sparse regression (STLSQ, BIC-selected λ) to discover the infection ODE system; (4) hospitalisation and ICU cases are predicted via Bayesian linear regression, refit on a rolling 60-day window; (5) one of three strategies refines the SINDy coefficients using recent data — local adjustment (7-day window), time-dependent adjustment (full history with regularisation), or neural-augmented adjustment (7-day window with a small neural network); (6) predictions are evaluated out-of-sample via walk-forward validation (14-day test windows, 2-day step). In the next section, we will look at some results using the proposed method.

## Results

Dataset illustration

The proposed model has been designed and developed based on Thuringia’s COVID-19 data, which covers 707 days of the pandemic. The raw reported data was processed and transformed into a time series dataset using the DataFrames.jl package [19] in the Julia programming language. This time-series dataset provides a detailed account of the progress of the epidemiological spread of the pandemic in the German state of Thuringia. For example, the smoothed version of infections for different age groups (left panel) and hospitalisations (and ICU cases) for the 60–79 age-group (right panel) are summarised in Fig 2. All reported results are based on this Thuringia dataset as the input.

**Fig 2:**
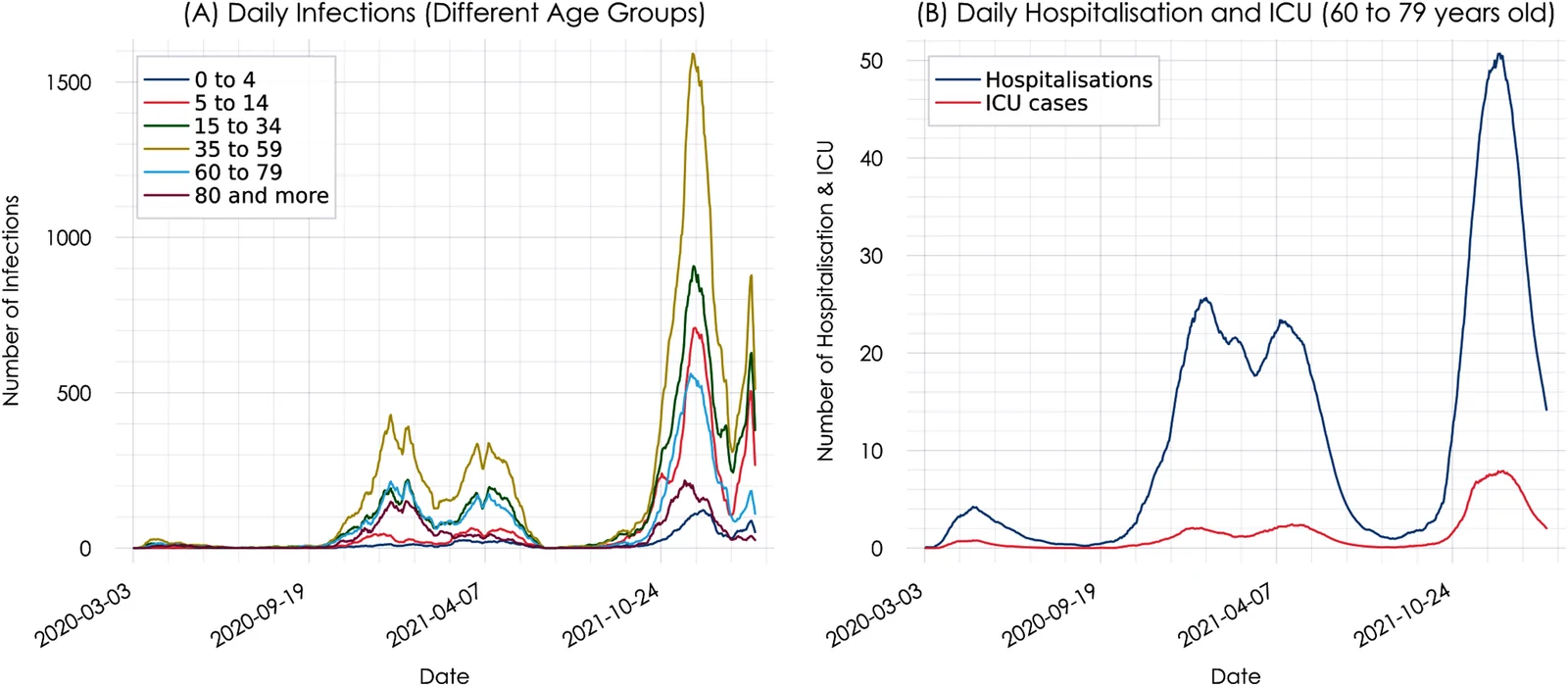
Some details of the Thuringia time-series dataset. The left panel (A) shows the measured infections per day for various age groups. The right panel (B) displays the hospitalisations and ICU cases (ages 60−79) attributed to COVID-19.

The plots shown in Fig 2 are based on weekly-averaged numbers. The same averaging process has been applied to the vaccinations data (Fig 3). The difference between the two plots shows the effect of weekly averaging (Fig 3, right panel) on noisy reported data (Fig 3, left panel).

**Fig 3:**
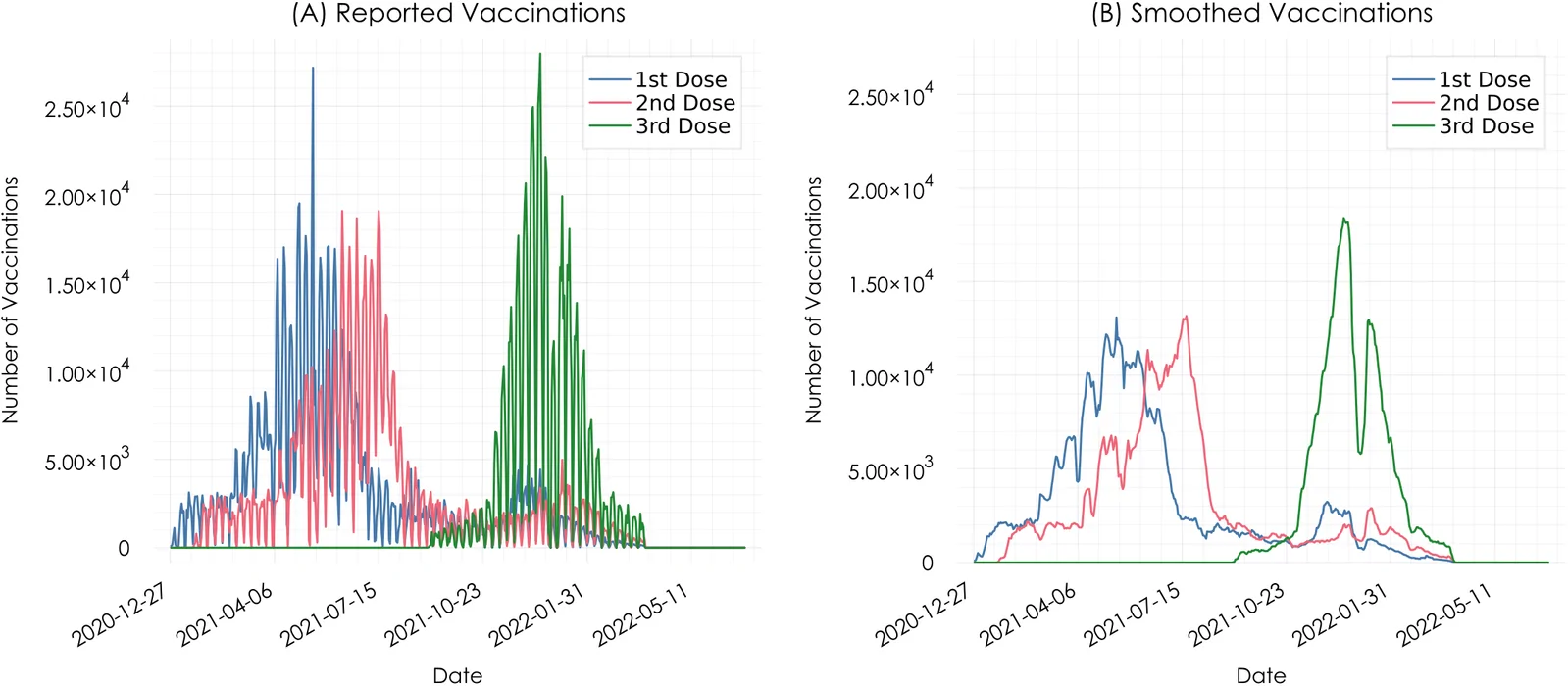
Vaccinations before and after averaging. On the left panel (A) the daily statistics are shown. On the right (B), only the weekly averaging is displayed.

Antibody and infectiveness

Two kernel functions produce two new features, antibody (known as *A*) and infectiveness (known as *y*) through convolution. Equations (3) and (4) in the previous section show how we calculate them. The antibody kernel spans k = 35 days with a *Beta(3, 4)* distribution, producing a response curve consistent with studies reporting that IgG levels peak approximately two weeks after vaccination and wane over subsequent months [20,21]. The infectiveness kernel spans k = 21 days with a *Beta(2.5, 4.5)* distribution, producing a right-skewed curve with peak infectiveness near the end of the first week. This is consistent with evidence that infectious virus shedding peaks within the first few days after symptom onset and can persist for up to two to three weeks [22,23]. The exact kernel parameters were selected within this literature-informed range based on model validation. We acknowledge that the final selection of the Beta distribution parameters (α, β) and kernel lengths (k) involves a degree of empirical tuning, and we note this as a reproducibility limitation, since the exact optimum may be dataset-specific. While the ranges were informed by published evidence, the specific values were chosen to optimise model performance on the Thuringia dataset. Both kernels were produced using the Julia programming language's Distributions.jl package [24]. Fig 4 shows the kernel functions and the results after convolution. All numbers are normalised to the [0,1] range. A closer look at the green lines on the Antibody1 plot (first dose) shows that despite having the same number of vaccinations on the 110^th^ and the 170^th^ day (see dotted green vertical lines), the level of antibody is much higher on the 170^th^ day due to the different previous vaccination history. The same delay rule applies to the 620^th^ and 660^th^ day on the infectiveness plot. These convolved signals will be further used in the mathematical models.

**Fig 4:**
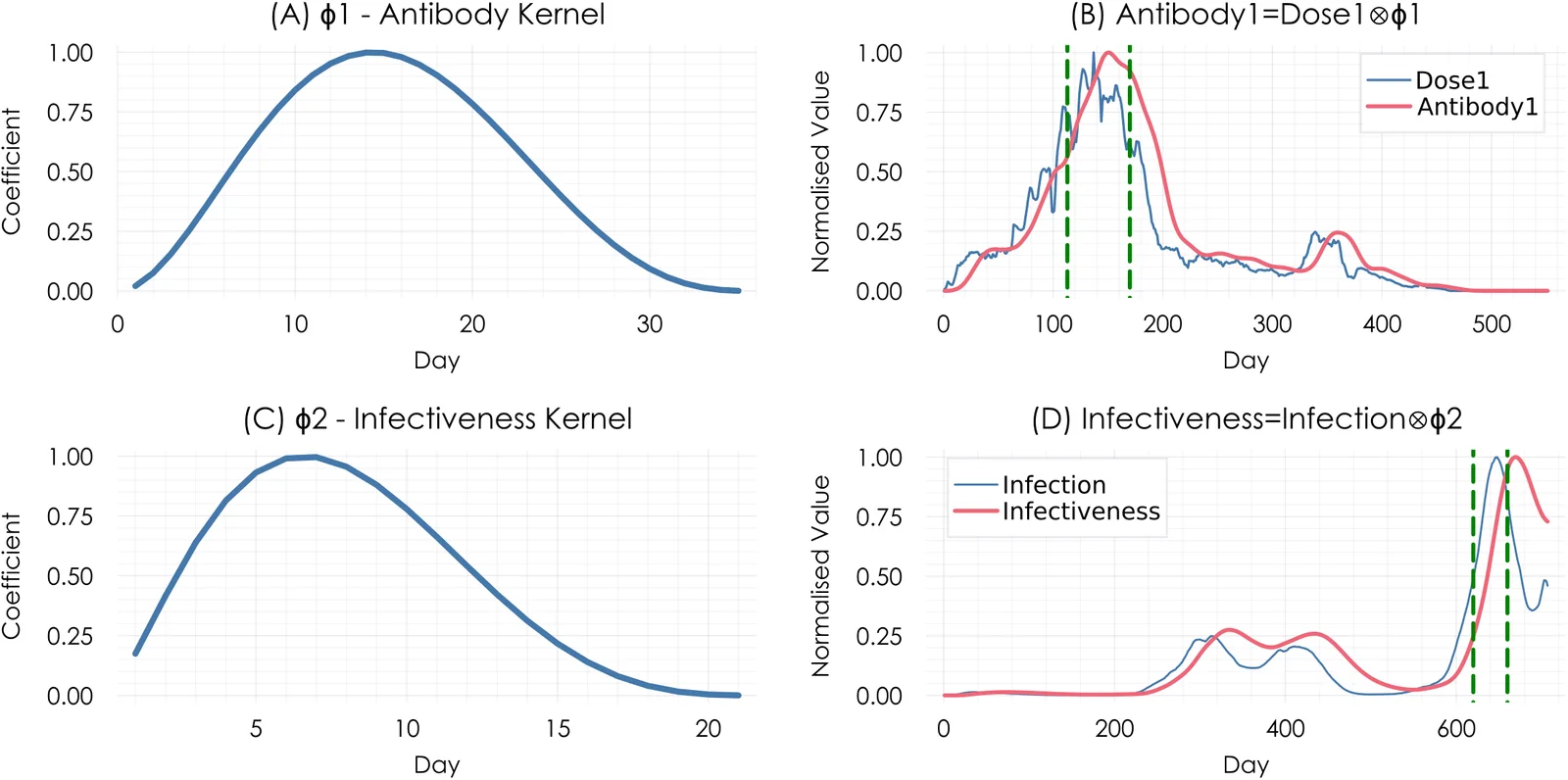
Antibody and infectiveness kernels. The left plots (A and C) show the kernel functions, while the right plots (B and D) illustrate the effect of convolution on vaccinations and infections using these kernels.

### Mathematical model for infections

The next step after pre-processing is developing a mathematical model for the data. Sparse Identification of Nonlinear Dynamic (SINDy) has been used to create a model of the infection dynamics. It is an automated model discovery approach. The parameters used in this research are specified in Table 2.

As evident from Table 2, the algorithm's input comprises two variables, infections and infectiveness (called *x, y*) as well as antibody (called *A*) as control signal. Antibody and infectiveness are the features extracted from the vaccinations and infections, as explained in the previous section. The range of *λ* that we use in the model discovery process is [10^*−5*^*, 10*^*−1*^]. *λ* is the regularisation parameter in the SINDy method, controlling the balance between fitting the observed data and sparsity. A higher value of *λ* encourages sparser models, meaning fewer terms are selected in the identified equations. Conversely, a lower value of *λ* allows for more terms to be included in the model, potentially capturing more complexity in the system dynamics. A quadratic library was chosen as the lowest-order basis able to represent saturation-type effects (e.g., the x² term in Eq 11) while keeping the candidate term count low enough for stable sparse regression on ~700 daily time points; higher-order bases were not pursued to avoid overfitting given the limited effective degrees of freedom in the smoothed time series. The DataDrivenDiffEq.jl package [25] was used to discover a mathematical model. The SINDy algorithm is part of this package. The STLSQ optimizer was run over λ values, with the data z-score normalised prior to fitting. DataDrivenDiffEq.jl automatically identified the Pareto-optimal model across this sweep by minimising the Bayesian Information Criterion (BIC), which penalises model complexity to balance sparsity against fit accuracy. According to the designed model, we can exclude any part of the data, make a model with the rest, and then run a prediction for the excluded part. For the prediction, we solve the differential equations numerically using the DifferentialEquations.jl package [26] in the Julia language.

Employing stochastic differential equations (SDEs) instead of ordinary differential equations (ODEs) allows for a more comprehensive representation of chaotic systems, such as the COVID-19 pandemic, by accounting for inherent uncertainties. While ODEs yield a single deterministic trajectory, SDEs generate a distribution of possible outcomes through iterative simulations. This is important for decision-making, because a single predicted trajectory does not convey how confident the model is about its forecast. The spread of the SDE ensemble provides a range of plausible outcomes, making it easier to assess risks and plan accordingly. The resulting spread reflects the system’s uncertainty, with the central tendency indicating the most probable evolution. Since the SINDy algorithm produces a deterministic ODE system, we augmented the discovered model with a diffusion term to formulate a corresponding SDE. The magnitude of this diffusion term was determined empirically through iterative testing; the value was selected to balance model stability and data fit. The resulting stochastic differential equation was solved using the Euler–Maruyama (EM) method from StochasticDiffEq.jl. Shaded bands in figures showing stochastic prediction (Figs 5, 8, 12, 13) represent the standard deviation across the 1,000 realisations. All projection results in this section were computed using the stochastic version of the model to account for uncertainty.

**Fig 5:**
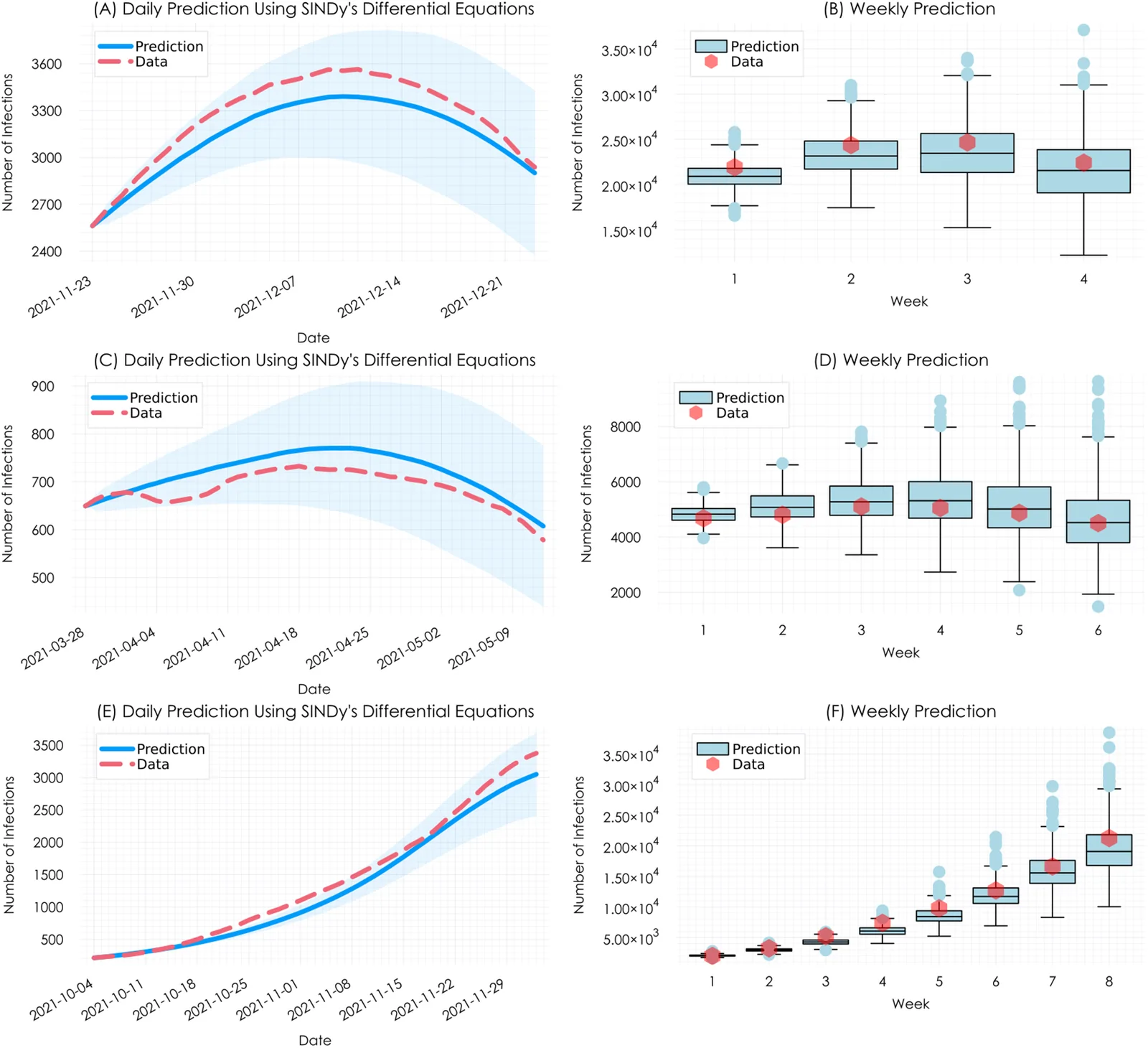
Daily and weekly predictions. The plots show predictions for one month (A and B), 45 days (C and D), and two months (E and F) across different time frames.

Fig 5 shows three examples of daily and weekly predictions after solving the SDE 1000 times. The plots on the top show a one-month prediction, the middle ones predict 45 days and the bottom plots make predictions for two months. A different range of dates was used in these examples. In the daily prediction plots, the most likely prediction is the blue line, but the ribbons specify other ranges of possibilities. Since the model was created using smoothed data, having a weekly prediction is necessary to make it helpful. The boxplots on the right side show the equivalent weekly predictions.

### Hospitalisations and ICU cases

Since there is a correlation between infections and hospitalisations (and ICU cases), linear regression was used to predict the number of hospitalisations (and ICU cases). As shown in the previous section, it is possible to predict the number of infections through the discovered mathematical model using SINDy. Now, we can use these infection numbers to forecast hospitalisations and ICU cases. To account for uncertainty, Bayesian statistics were used. To ensure the model captures the most recent trends in the correlation between infections and hospitalisations (and ICU cases), only the 60 data points prior to the prediction day were used to train the Bayesian model. A 60-day window was chosen as a compromise between capturing enough data points for stable posterior estimation of the three regression parameters (α, β, σ) and remaining short enough to track potential drift in the infection–hospitalisation relationship (e.g., due to changing variant severity or vaccination coverage) rather than averaging over the entire pandemic period. A probabilistic regression was applied to predict the number of hospitalisations (and ICU cases). The posterior distribution (for hospitalisations) after 1,000 iterations with the No-U-Turn Sampler (NUTS) is shown in Fig 6. It is carried out according to Equation (6). The Turing.jl package [27] was used for probabilistic programming. The first 500 iterations were used to stabilise the stochastic model. *α*_*h*_, *β*_*h*_ and *σ*_*h*_ in the plots represent intercept, slope, and observation noise of the hospitalisations, respectively. Convergence diagnostics confirmed good mixing, with Gelman–Rubin statistics (R̂) values below 1.01 and effective sample sizes (ESS) above 2000 for all parameters.

**Fig 6:**
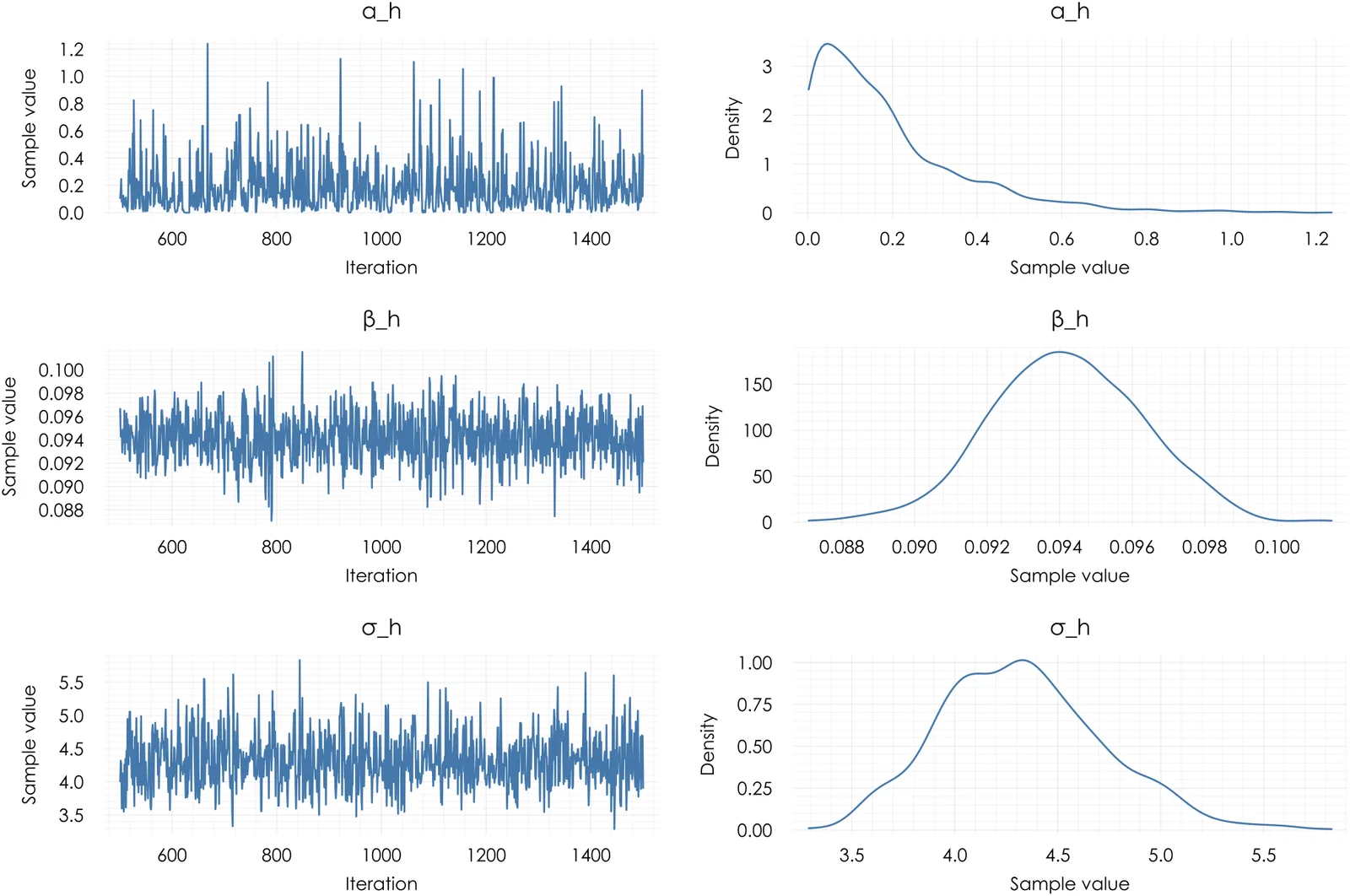
Posterior distributions of regression parameters. Probability Distribution of intercept (α_h_), slope (β_h_) and observation noise (σ_h_) after 1000 iterations (after 500 warm-up iterations) using NUTS. All parameters showed good convergence, (R̂ < 1.01) and effective sample sizes above 2000.

Using the *α*_*h*_, *β*_*h*_ and *σ*_*h*_ (and also the *α*_*u*_, *β*_*u*_ and *σ*_*u*_) probability distributions, we can calculate a probabilistic line to visualise the accuracy of regression in the Thuringia dataset. Fig 7 shows these lines (top panel) as well as the predictions for hospitalisations and ICU cases (bottom panel).

**Fig 7:**
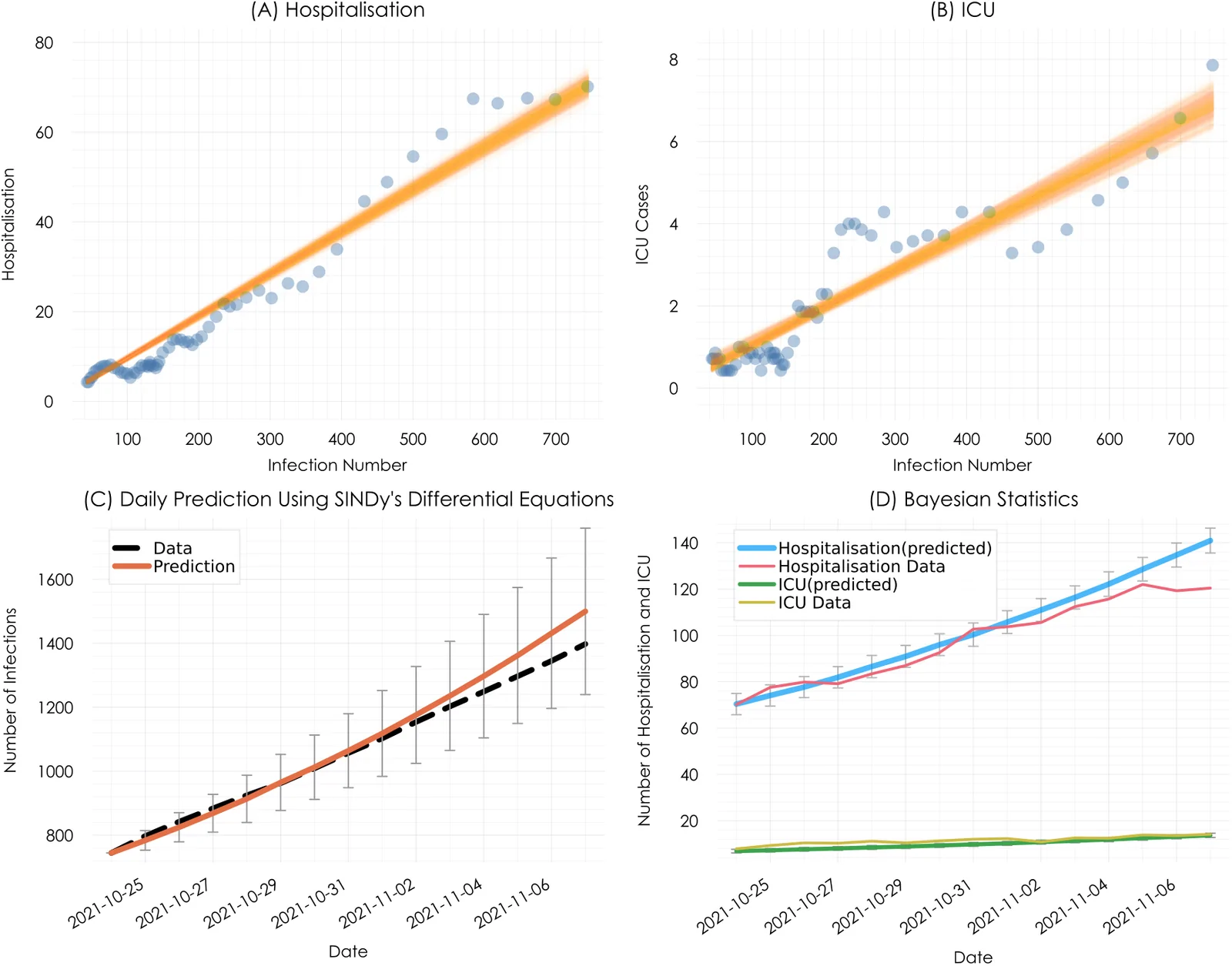
Using Bayesian statistics to predict hospitalisations and ICU cases. The panels on top (A and B) show the probabilistic lines. The orange line indicates the fit and +/ − one sigma, and the panels on the bottom (C and D) show the predictions.

### A useful query

Since the control signal in the mathematical model is the antibody (obtained from vaccinations), we can feed the model with any arbitrary vaccination rate to see what happens in different scenarios. For example, the influence of the vaccination status on the prediction can be studied by performing simulations with modified parameters. The 35-day window used to modify vaccination inputs in each scenario matches the antibody kernel length (k = 35 days) used in the convolution step (see Methods), so that the modified vaccination history is fully reflected in the antibody signal by the start of the prediction period. In Fig 8, there are four different scenarios:

**Fig 8:**
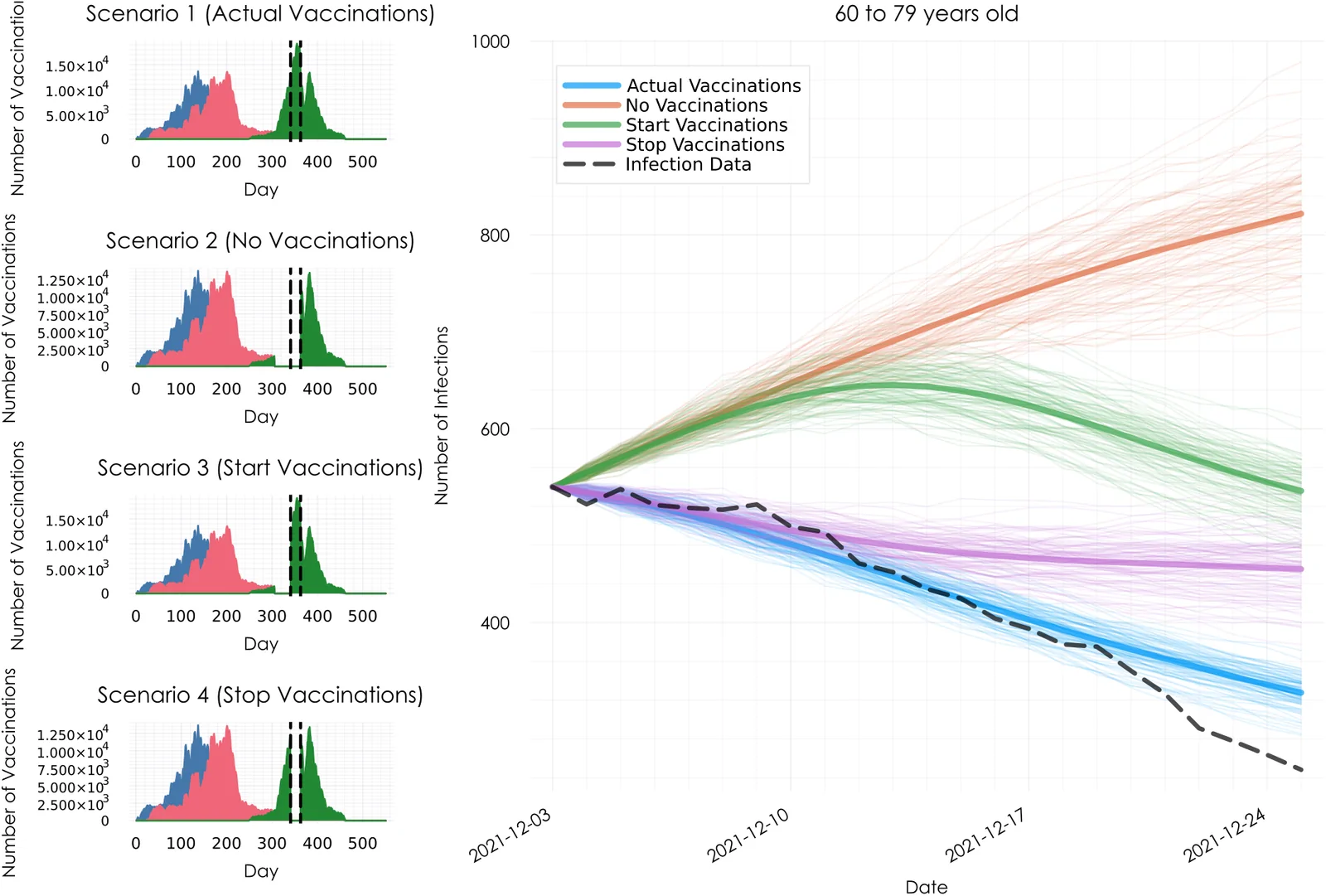
Predictions under different vaccination scenarios. Left panels show vaccination inputs (blue: 1st dose, red: 2nd dose, green: 3rd dose) with black vertical lines indicating the prediction window. Right panel shows predicted infections for each scenario against observed data.

1. actual vaccinations: prediction with actual vaccinations, which is relatively compatible with the data.
2. no vaccinations: no one is getting vaccinated from 35 days before the prediction’s start day until the end of the prediction dates, which shows a sharp increase in the infections. According to the antibody kernel function (Equation 4), this scenario means zero antibodies during the prediction days.
3. start vaccinations: no further vaccinations from 35 days before the prediction date up to the start of the prediction dates, then just from the first day of prediction, vaccinations start at the actual rate (equal to actual vaccinations). The result of this scenario initially shows an increase in the infections, but after a week, it starts to decrease because of the gradual accumulation of the antibody.
4. stop vaccinations: this scenario has the actual vaccination rate before the prediction day, but after that, no further vaccinations are given during the prediction dates. The result initially shows a similar result to the scenario one, but gradually, the infections increase due to the lack of vaccinations. Fig 8 illustrates how our model uses the antibody (control signal) to incorporate the vaccinations history.

The comparison of the results across different scenarios suggests that, according to the model, changes in vaccination rates are associated with substantial differences in predicted infection trajectories. However, as the model is derived from observational data, these projections should not be interpreted as causal conclusions. From a practical perspective, this type of scenario analysis could support decision-making during a pandemic. For example, in a research or planning context, such a framework could be used to explore the potential consequences of delaying a vaccination campaign, or to assess whether a given vaccination rate might be sufficient to prevent the next wave. Since the model runs quickly, such analyses can be repeated as new data becomes available, providing a basis for exploratory intervention planning.

### Coefficient optimisation

The governing differential equations of the pandemic are extracted from data using SINDy. These equations include several coefficients, which are influenced by external factors such as public health interventions, medical breakthroughs, and changes in virus strain over time. The model does not attribute coefficient changes to these factors individually; rather, it captures their aggregate, unresolved effect on the coefficients, without distinguishing which factor is responsible for a given shift. Optimising these coefficients helps us understand the effects of such factors on the pandemic and identify moments when significant changes occurred. We refer to the coefficients in the original ODE extracted by SINDy as global coefficients. As explained before, we use three approaches to optimise the global coefficients:

- Local coefficient adjustment
- Time-dependent coefficient adjustment
- Neural-augmented ODE adjustment

In this part, we want to show how practically effective each approach is. Table 3 shows the parameters used in optimisation for the two first approaches (local coefficient adjustment and time-dependent coefficient adjustment). All of the parameters are identical except the penalty function (see Equations 7 and 8) and the fact that in the local coefficient adjustment, we used seven days of data before the prediction day to adjust the coefficients and then made predictions, but in the time-dependent coefficient adjustment, we optimised the coefficients for every single day during the pandemic and then used the prediction start day’s coefficients to make predictions. In the local coefficient adjustment, the DiffEqParamEstim.jl package [28] was used to optimise the coefficients, but in the time-dependent coefficient adjustment, we used the Optim.jl package [29].

To check the efficiency of the approaches, we considered a date on which the global coefficients perform poorly, i.e., producing high residuals. An ideal test case is a day in the middle of a sharp surge in infections, when authorities typically intervene with restrictions. Fig 9 shows the observed data and predictions using three different coefficient sets. The vertical line is the start day of the prediction, and the seven days before this line were used for the local coefficient adjustment. As is evident from Fig 9, the prediction based on the global coefficients does not fit the observed data well. After the local coefficient adjustment, the prediction is better, but still deviated from the data. The third approach is the time-dependent coefficient adjustment, in which the model captures the observed trend more closely, suggesting that it accounts for time-varying factors more effectively.

**Fig 9:**
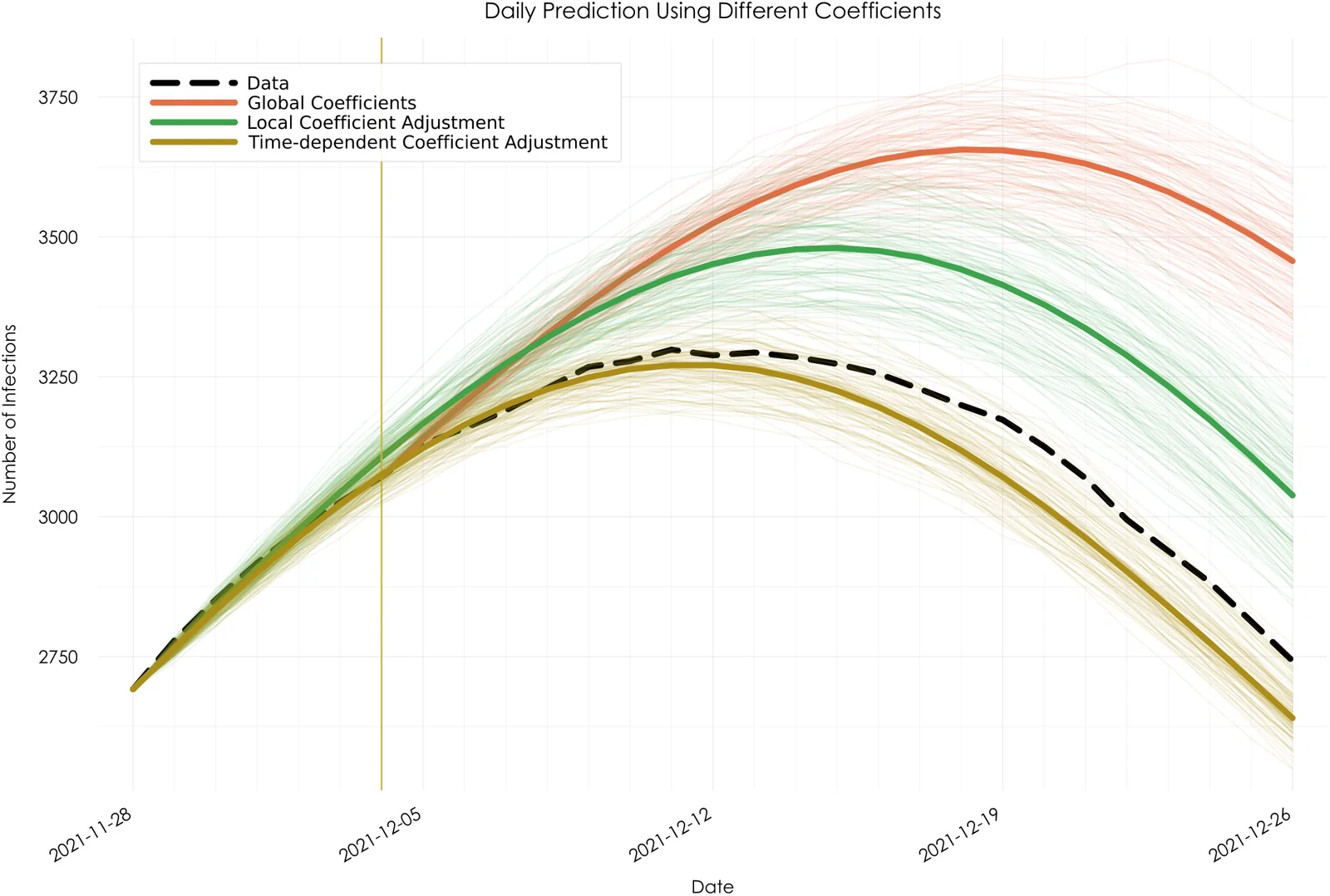
Daily infection predictions using three coefficient strategies. global (orange), locally adjusted (green), and time-dependent (yellow). The dashed black line shows observed infections. The red vertical line marks the prediction start date.

The third approach is the neural-augmented ODE adjustment, implemented using the universal differential equation (UDE) framework. Table 4 shows the parameters used in the optimisation process. This optimisation is carried out in two steps. The adaptive moment estimation (ADAM) is used in the first step with a learning rate 0.001. In the second step, the output of ADAM will be further optimised using Broyden-Fletcher-Goldfarb-Shanno (BFGS). The RBF activation was chosen over standard choices (e.g., tanh, ReLU) because its localized response is well suited to approximating smooth, bounded corrections to the ODE dynamics over the short optimisation window. The Lux.jl package [30] was used to create the neural network part and the Optimization.jl [31] package was used to optimise the UDE.

Fig 10 shows the optimisation results for the same time period as Fig 9. The vertical line is the start day of the prediction. The days before this line (seven days) were used to train the neural network in the UDE. The prediction is closely aligned with the observed data, indicating that external factors have been well captured. To test robustness, we simulated with ±5% variation in the initial infection values. The resulting predictions remain stable and consistent, as shown by the shaded blue region.

**Fig 10:**
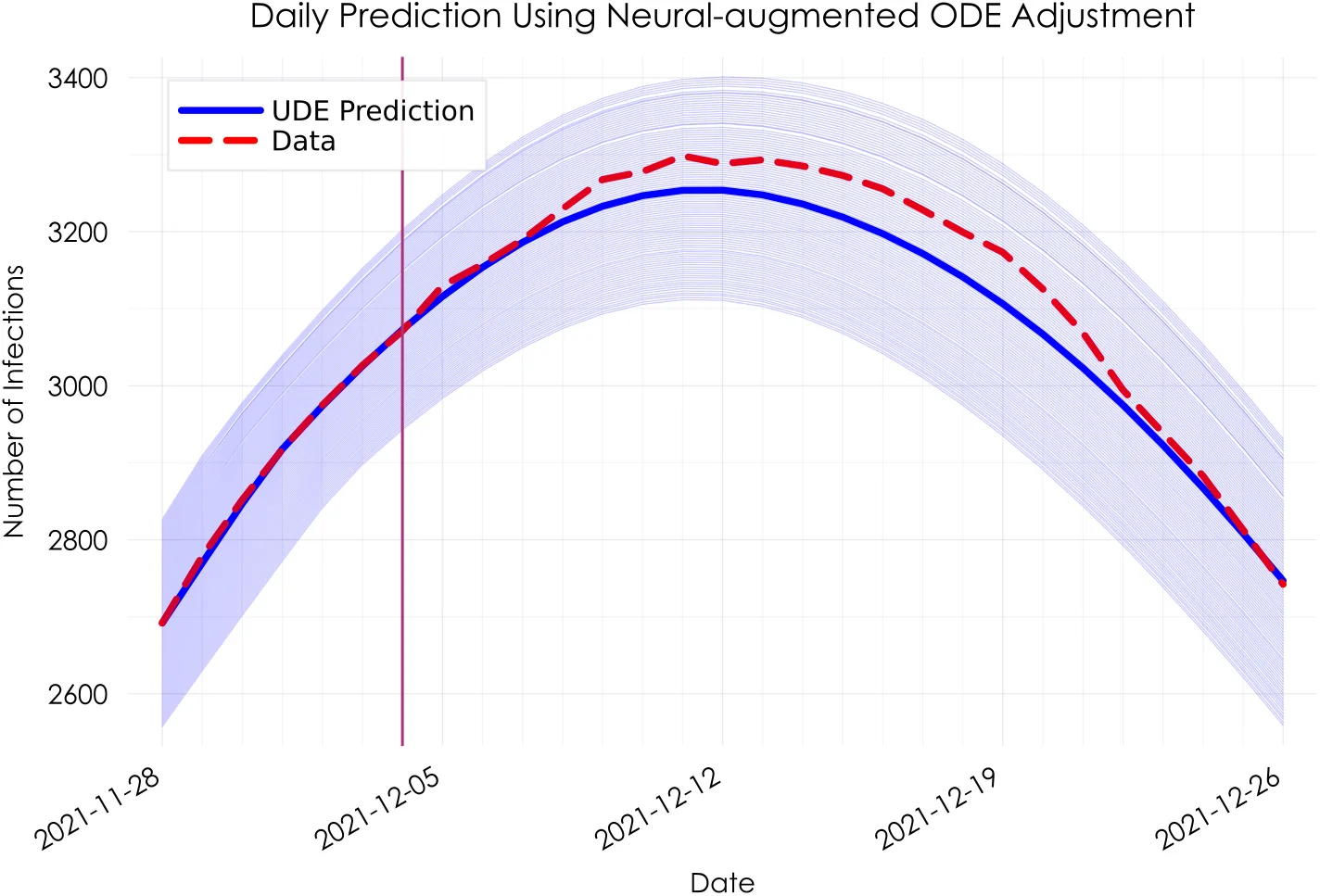
Infection prediction with neural-augmented ODE approach. The blue line shows the prediction, the shaded area reflects ±5% variation in initial values. The red vertical line marks the prediction start date.

### Model evaluation and validation

After employing three different optimisation methods, we have four options for prediction, global coefficients, local coefficient adjustment, time-dependent coefficient adjustment, and neural-augmented ODE adjustment. To assess all of them, we utilised the walk-forward validation (WFV) method, which functions similarly to cross-validation in time-series data [32]. During each evaluation round, a data window is segmented as test data, initially positioned at the start and progressively moved forward through the dataset. Our window spanned 14 days, or two weeks, and shifted forward by two days in each step. Fig 11 displays the average percentage of the absolute residuals. The results indicate that the time-dependent coefficient adjustment captures temporal external factors most effectively, although the neural-augmented ODE adjustment performs slightly better towards the end of the second week. A key observation is that, across all optimisation methods, the residuals initially improve but then gradually increase, nearing the performance of the global coefficients. This trend suggests that these optimisation methods effectively capture temporal factors, making them well-suited for short-term predictions.

**Fig 11:**
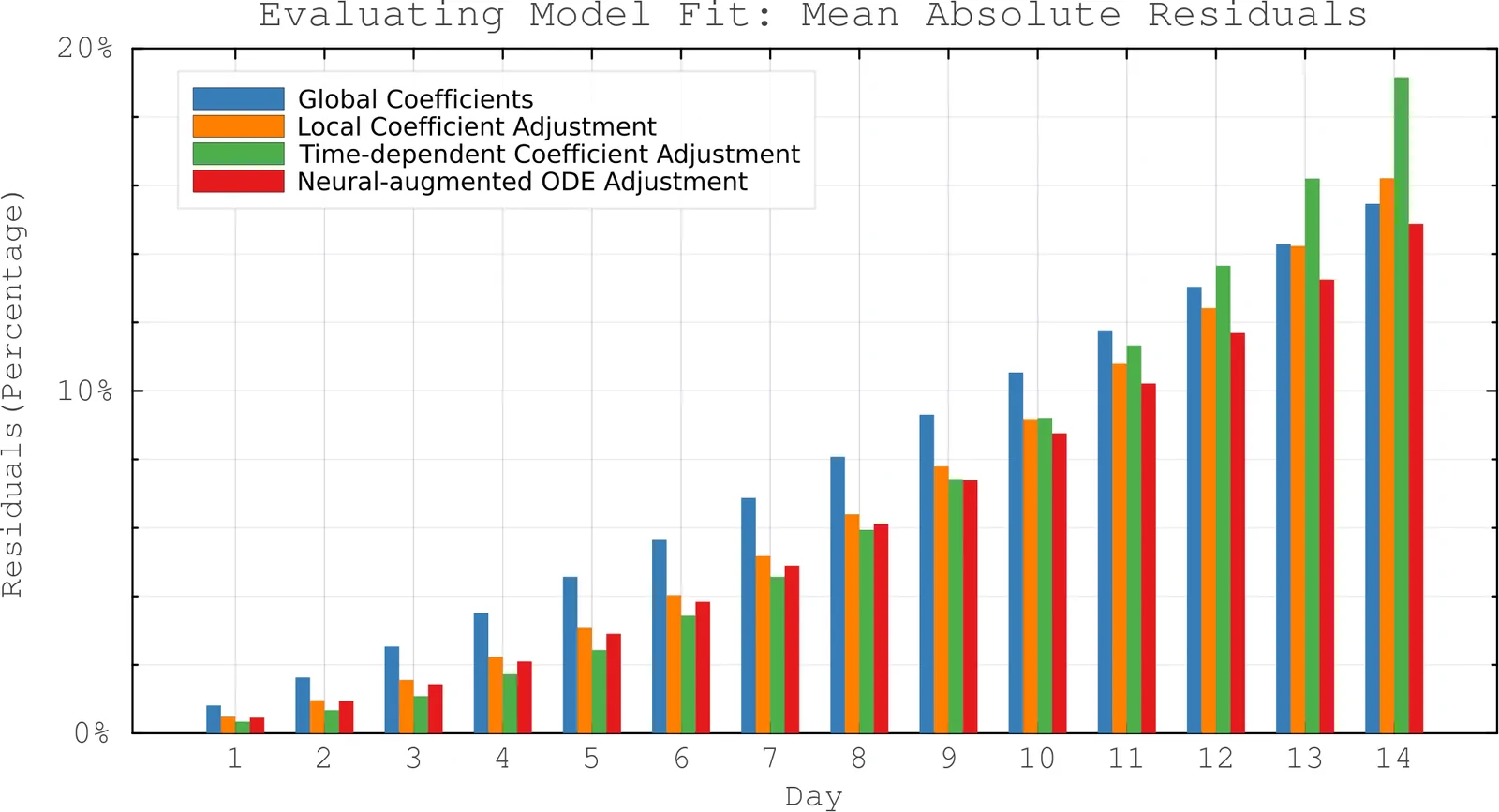
Mean absolute residuals (%) over a 14-day walk-forward validation for four prediction strategies. Time-dependent and neural-augmented approaches yield lower residuals, with all methods performing best in the short term.

Table 5 presents three key metrics—*RMSE* (Root Mean Squared Error)*, MAE* (Mean Absolute Error)*, and R*^*2*^ (coefficient of determination)—of the predictive models over time intervals of three, seven, ten, and fourteen days, calculated based on the number of infections. Since the walk-forward validation uses overlapping test windows (14-day window with a 2-day step), adjacent folds share test data, introducing dependence between error estimates. To account for this, 95% confidence intervals were computed using a block bootstrap procedure (block size = 7), which groups dependent folds together to produce reliable uncertainty estimates. *RMSE* and *MAE* are standard measures of prediction error, with RMSE giving higher weight to larger errors due to squaring, while *MAE* provides a more uniform assessment of average error magnitude. *R²* indicates how well the model explains the variance in the observed data, with values closer to 1 representing better fit. The results indicate that the time-dependent coefficient adjustment approach achieves the highest accuracy, capturing over 87% of the predictive variance by the first week (*R*^*2*^ = *0.87*) and maintaining good performance after ten days (*R*^*2*^ = *0.73*). However, towards the two-week horizon, the neural-augmented ODE adjustment demonstrates superior outcomes, as shown in Figs 9 and 10, offering more reliable predictions in scenarios of higher infection rates. Given that daily infection rates in Thuringia can reach up to 4,000 cases, the observed values of RMSE and MAE suggest reasonable accuracy for short-term forecasts, particularly within the first ten days. While the time-dependent coefficient adjustment shows the highest point estimates for R² at most horizons, the 95% bootstrap confidence intervals in Table 5 overlap substantially between methods at longer horizons. At day 14, the R² intervals for the time-dependent adjustment [0.07, 0.83] and local coefficient adjustment [−0.22, 0.85] overlap almost entirely, and the global-coefficient interval extends to −0.39 at its lower bound. This indicates that the observed differences in point performance between optimisation methods are not statistically distinguishable beyond approximately 7–10 days, and predictive performance for all methods degrades substantially at longer forecast horizons.

**Table 5:** Comparative performance metrics of predictive approaches at four forecast horizons, with 95% bootstrap confidence intervals.

Day	Metric	Global coefficients	Local coefficient adjustment	Time-dependent coefficient adjustment	Neural-augmented ODE adjustment
3^rd^	**R** ^**2**^	0.75 **[0.59, 0.89]** 15.68 **[8.92, 22.21]** 11.96 **[6.68, 17.01]**	0.91 **[0.86, 0.97]** 9.39 **[4.46, 14.65]** 7.06 **[3.37, 10.96]**	0.96 **[0.92, 0.98]** 6.73 **[4.13, 8.93]** 4.97 **[3.04, 6.59]**	0.92 **[0.87, 0.96]** 9.14 **[4.81, 13.61]** 6.86 **[3.58, 10.23]**
	**RMSE**				
	**MAE**				
7^th^	**R** ^**2**^	0.58 **[0.28, 0.82]** 48.65 **[28.48, 69.81]** 39.28 **[23.04, 56.76]**	0.81 **[0.59, 0.95]** 33.23 **[15.69, 56.78]** 25.91 **[12.22, 43.84]**	0.87 **[0.83, 0.94]** 26.95 **[16.23, 38.63]** 20.35 **[12.39, 28.68]**	0.83 **[0.72, 0.92]** 31.41 **[18.46, 47.54]** 24.51 **[14.36, 36.9]**
	**RMSE**				
	**MAE**				
10^th^	**R** ^**2**^	0.46 **[0.04, 0.78]** 77.8 **[48.15, 110.21]** 63.14 **[38.57, 89.86]**	0.68 **[0.31, 0.91]** 60.25 **[29.85, 104.11]** 46.6 **[23.06, 80.1]**	0.73 **[0.6, 0.89]** 54.63 **[31.14, 84.19]** 40.61 **[23.87, 61.28]**	0.71 **[0.49, 0.88]** 57.22 **[30.17, 90.11]** 44.2 **[23.18, 69.39]**
	**RMSE**				
	**MAE**				
14^th^	**R** ^**2**^	0.31 **[−0.39, 0.75]** 119.35 **[76.09, 170.92]** 97.22 **[61.4, 140.76]**	0.45 **[−0.22, 0.85]** 107.27 **[56.47, 181.71]** 82.23 **[42.9, 138.39]**	0.39 **[0.07, 0.83]** 112.52 **[57.08, 182.89]** 81.92 **[43.22, 128.9]**	0.51 **[0.01, 0.81]** 100.76 **[57.99, 156.76]** 77.69 **[44.3, 120.0]**
	**RMSE**				
	**MAE**				

### Optimisation insights

As outlined above, we employ three distinct strategies to optimise the global coefficients. This raises the question: why are multiple methods necessary, rather than relying on a single approach? The answer lies in the fact that each optimisation strategy offers its own advantages and is suited to different scenarios. While the neural-augmented ODE adjustment generally yields the most favourable outcomes for two-week prediction, the local coefficient adjustment and the time-dependent coefficient adjustment offer their own advantages.

Following the time-dependent coefficient adjustment, we obtain time-dependent coefficient values, represented as an array denoted as *c*, which encapsulates the optimal coefficients for each pandemic day. Leveraging these coefficients, we not only enhance prediction accuracy by utilising the coefficients specific to each day (*c*_*i*_ for date *i*) but also facilitate scenario analysis. We can simulate the potential outcomes of interventions initiated from the respective dates by employing coefficients from specific dates, such as during a lockdown. This provides valuable insights into the effectiveness of interventions throughout different stages of the pandemic. Fig 12 demonstrates the application of the time-dependent coefficient adjustment. The vertical line marks the prediction starting day, with three predictions presented: the orange and green lines represent predictions made using the global coefficients and the time-dependent coefficient adjustment, respectively. It is evident from Fig 12 that the time-dependent coefficient adjustment yields more accurate predictions than the global coefficients. The bottom line depicts the potential effects of an intervention. By utilising coefficients observed during previous lockdowns (April–June 2021), it illustrates the potential outcome if a lockdown were to be initiated from the prediction starting day.

**Fig 12:**
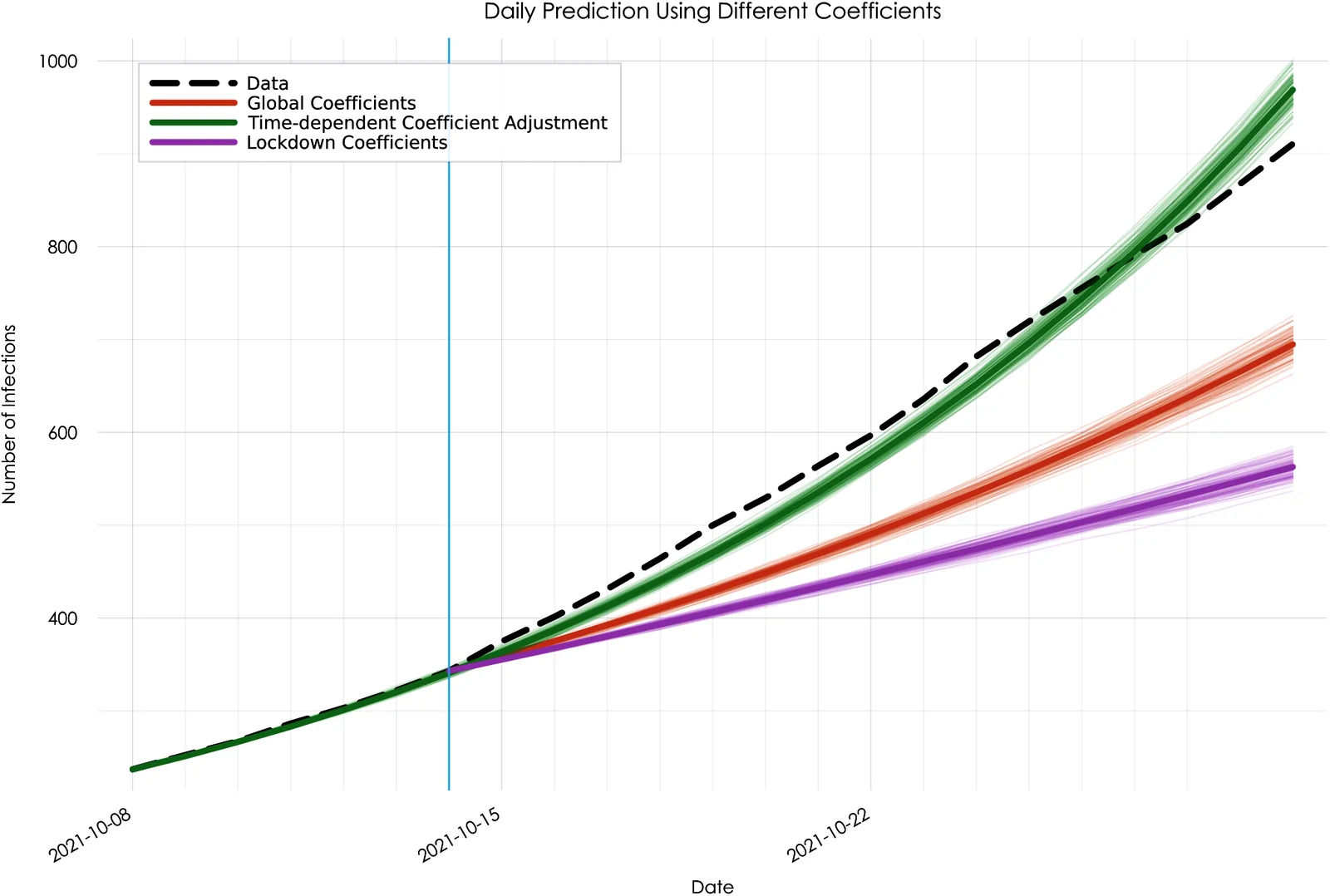
Daily infection predictions using different coefficient strategies. Global coefficients (orange), time-dependent coefficients (green), and a lockdown scenario using past restriction-period coefficients (purple) are compared against observed infections (dashed black line).

The local coefficient adjustment is another approach we used to optimise the global coefficients. While the time-dependent coefficient adjustment and the neural-augmented ODE adjustment yield superior results overall, our study reveals a notable advantage of the local coefficient adjustment in scenarios with extremely low infections. Throughout our analysis of the Thuringia pandemic dataset, where infection numbers typically ranged from 0 to 4000, we encountered challenges when daily cases dropped below 50. In such instances, global coefficients often faltered, yielding unreliable predictions. However, through the application of the local coefficient adjustment, we achieved efficient calibration of coefficient values, enabling the model to forecast future trends accurately despite minimal infection rates. As illustrated in Fig 13, the local coefficient adjustment visibly tracked the observed low-incidence trend more closely than the global coefficients, which struggled when daily cases dropped below 50, demonstrating its potential value as a complementary tool in these circumstances. Fig 13 shows the model's outputs using the global coefficients and the local coefficient adjustment. The vertical line shows the prediction starting day. The seven days before this line were used in the optimisation process.

**Fig 13:**
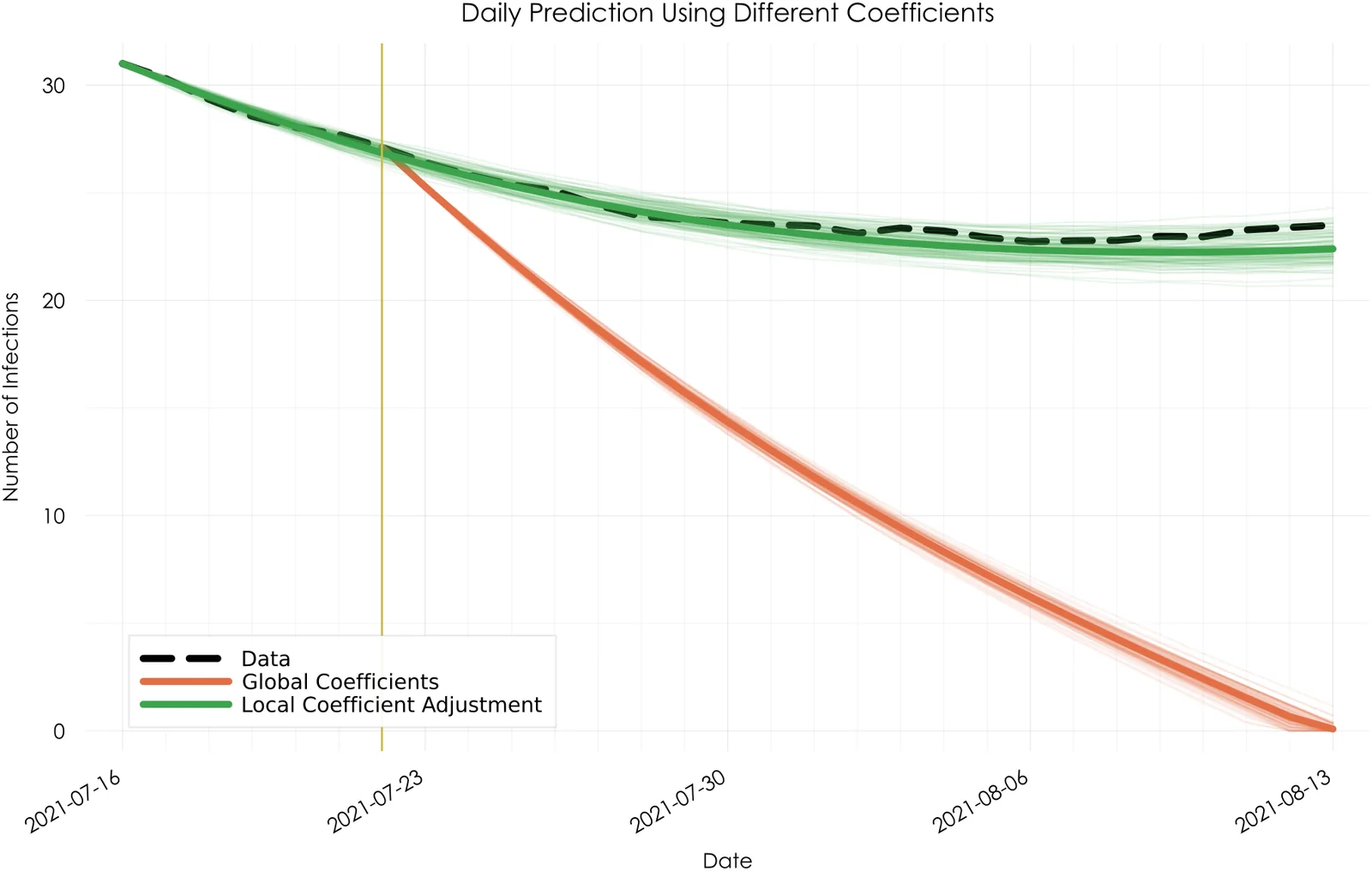
Daily infection predictions in a low-incidence scenario. Global coefficients (orange) fail to capture the trend, while locally adjusted coefficients (green) follow the observed data (dashed black line, below 50 cases/day) closely.

### What we learn from the model

By looking at this differential equation extracted by SINDy (Equation 11) we can gain a better insight into the dynamics of the pandemic. Here, *x* represents the daily number of infections, *y* represents the infectiveness (a convolution of *x*), and *A* shows the antibody effect from the vaccinations (convolution of the vaccinations *v*).


$$\begin{array}{c} \begin{array}{c} \left\{ \;\;\begin{array}{c} \frac{dx}{dt}=\text{0}.\text{1}\text{0}\text{3}x-\text{0}.\text{0}\text{7}\text{6}y-\text{0}.\text{1}\text{2}Ax+\text{0}.\text{0}\text{6}\text{4}Ay\;\\ \frac{dy}{dt}=\text{0}.\text{0}\text{4}\text{9}x-\text{0}.\text{0}\text{5}\text{4}y+\text{0}.\text{0}\text{5}\text{2}Ax-\text{0}.\text{0}\text{2}\text{1}Ay-\text{0}.\text{0}\text{0}\text{4}x^{\text{2}} \end{array}\;\right. \end{array} \end{array}$$ (11)

This model, derived directly from the data, highlights key dynamics of the pandemic. The first equation suggests that while infections (*x*) grow naturally at a base rate (0.103), they are suppressed by infectiveness (*−0.076y*) and further mitigated by vaccination (*−0.12Ax*). The relatively small suppressing effect of infectiveness might suggest a decrease in the susceptible population. Interestingly, the term *Ay* positively contributes to the infections, potentially reflecting partial vaccine efficacy.

The second equation captures the dynamics of infectiveness (*y*). Since it is the convolution of the infections, it grows according to the infections (*0.049x*) and then decays over time (*−0.054y*). Vaccination in this equation exhibits a dual effect: On the one hand, it suppresses infectiveness (*−0.021Ay,*) which is the direct biological effect of the vaccinations, on the other hand, the positive contribution of the Ax term (*0.052Ax*) represents an indirect, data-driven association rather than a direct biological mechanism. Several plausible explanations exist: it may reflect behavioural changes among vaccinated individuals (e.g., reduced adherence to protective measures), or it may capture the effect of policy relaxation (e.g., easing of restrictions) that coincided with rising vaccination rates. Since SINDy identifies statistical associations from observational data, disentangling these effects is beyond the scope of the current model. The opposite signs of the Ax term in the two equations reflect the different roles of the antibody variable in the system. While the negative contribution in the infection equation is consistent with the protective effect of antibodies, the positive contribution in the infectiveness equation captures indirect, data-driven effects present in the observed dynamics. Notably, the magnitude of the negative term exceeds the positive contribution, indicating an overall protective effect of antibody levels. The nonlinear term *−0.004x*^*2*^ suggests a saturation effect at high infection levels, indicative of population-level herd immunity, and it may also reflect underreporting when health institutions are overwhelmed. To deepen our understanding of the model’s dynamics, we now turn to its sensitivity analysis.

To verify that the discovered model structure is insensitive to the length of smoothing window, we repeated the full SINDy model discovery pipeline using two days shortened and two days prolonged averaging window lengths for comparison. Table 6 shows the coefficients of the terms that were consistently selected by the algorithm. The same set of active terms was recovered in every case, with identical signs, confirming that the structural form and the epidemiological interpretation of the model are robust to this pre-processing choice. In particular, the infection growth coefficient in the first equation, which is the dominant driver of the dynamics, remained virtually unchanged across such changes to the averaging window.

**Table 6:** Comparison of coefficients for the terms consistently identified by the SINDy algorithm across different smoothing windows.

Equation	Term	Shorter window	Baseline window	Longer window
dx/dt				
	**x**	0.104	0.103	0.099
	**y**	−0.073	−0.076	−0.082
	**Ax**	−0.151	−0.12	−0.091
	**Ay**	0.073	0.064	0.059
dy/dt				
	**x**	0.054	0.049	0.046
	**y**	−0.06	−0.054	−0.047
	**Ax**	0.059	0.052	0.035
	**Ay**	−0.02	−0.021	−0.02
	**x** ^**2**^	−0.007	−0.004	−0.002

In addition to the smoothing sensitivity analysis, we also investigated the sensitivity of the model to the choice of the Beta distribution parameters used in the convolution kernels. To this end, we varied the shape parameters of the infectiveness and antibody kernel around the baseline configuration and repeated the full SINDy model discovery pipeline for each case. Fig 14 shows the resulting kernel shapes together with the corresponding coefficients of the discovered differential equations. The results indicate that moderate variations in the kernel parameters have a limited impact on the identified model. In all tested configurations, the same set of active terms was recovered, with consistent signs and similar coefficient values. This demonstrates that the discovered model structure is robust to the specific choice of the Beta distribution parameters.

**Fig 14:**
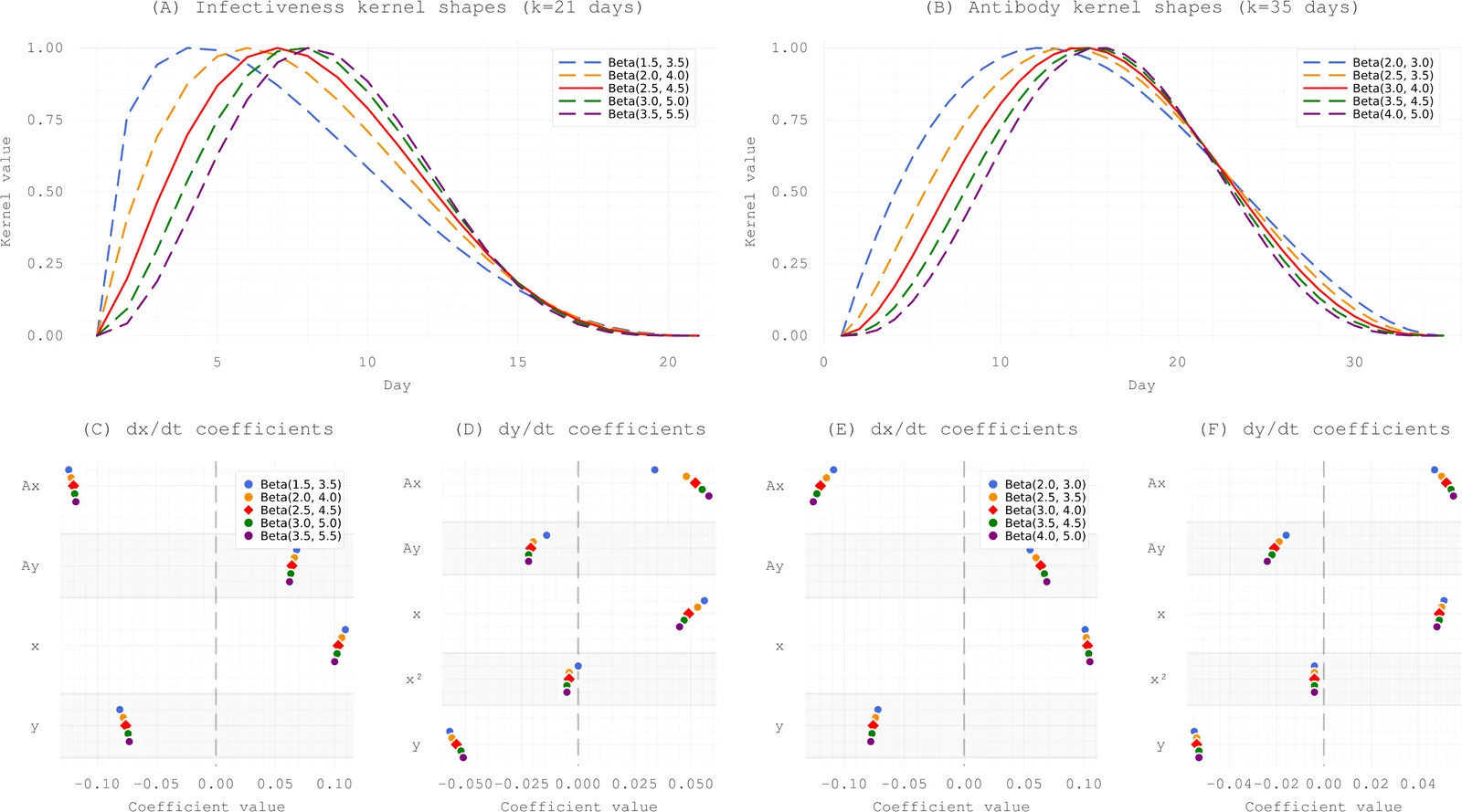
Sensitivity of the discovered model to kernel parameter variations. (A–B) Infectiveness (k = 21 days) and antibody (k = 35 days) kernel shapes under different Beta distribution parameters. (C–D) Corresponding dx/dt and dy/dt coefficients for infectiveness kernel variations. (E–F) Coefficients for antibody kernel variations. Coefficient values remain stable across all configurations.

The sensitivity analysis of our model (Fig 15) shows that the infection coefficient, which scales with the number of active infections, has a strong effect on how the outbreak progresses. This impact is most noticeable at the beginning or middle of a new wave, where even a small reduction in the number of infectious individuals can lead to a significant drop in future cases. Within the model, this suggests that isolating infectious individuals could help control viral spread early on by reducing their contribution to transmission. On the other hand, during the decline phase, changes in the infection rate have less effect, and at the peak of a wave, the model becomes unstable under sensitivity analysis, making predictions less reliable.

**Fig 15:**
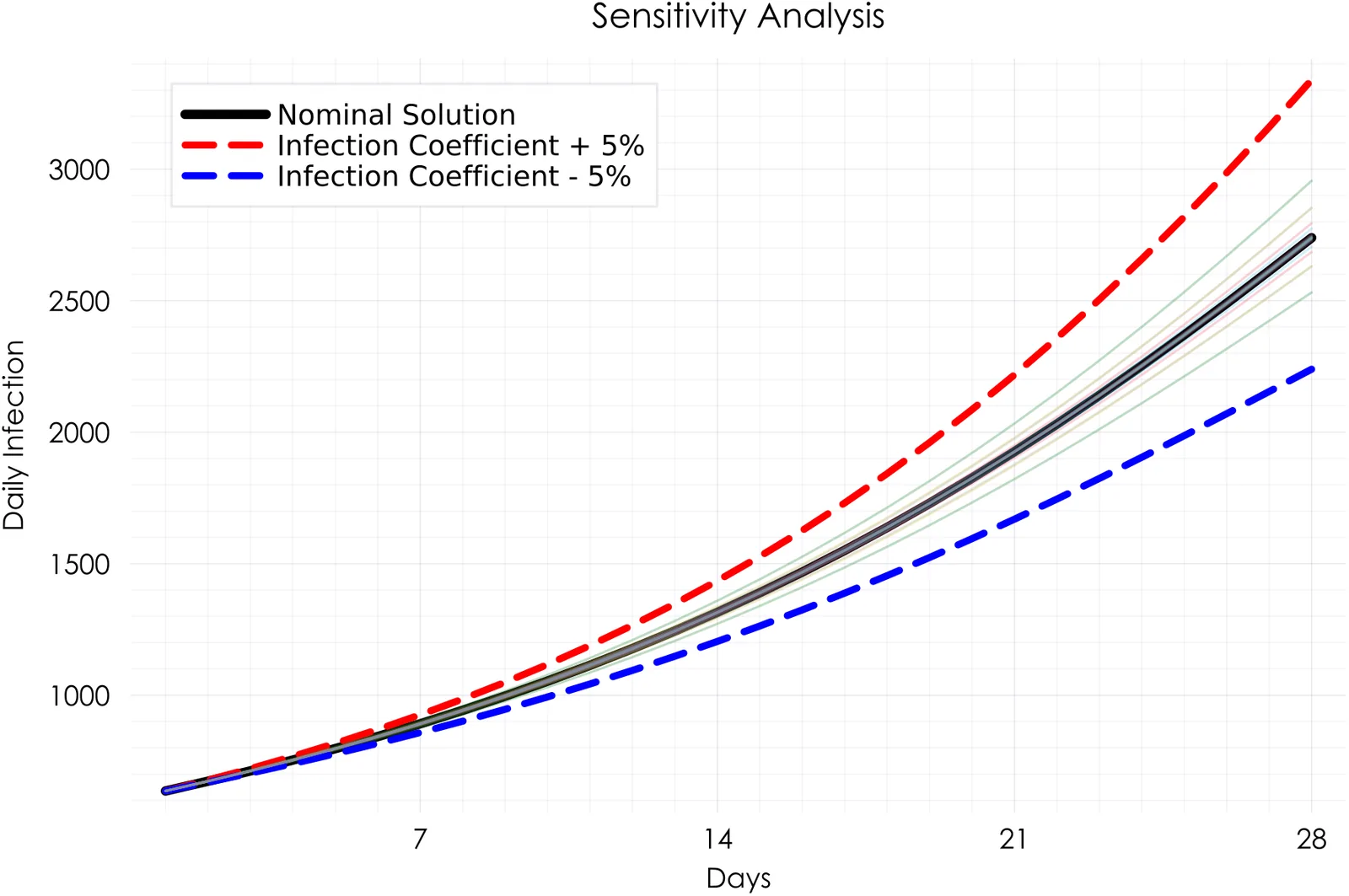
Sensitivity analysis of ODE model coefficients. The black line shows the nominal prediction. Red and blue dashed lines show ±5% perturbation of the infection coefficient; faded lines show similar perturbations of other coefficients. The infection coefficient produces the largest output variation.

## Discussion and conclusion

In conclusion, this study demonstrates that the governing equations of a real-world epidemic can be discovered directly from surveillance data, rather than assumed a priori, while retaining the interpretability of a mechanistic model. This automated discovery, and the transparency of the resulting equations and their coefficients, is the central contribution of this work — distinct from the predictive capability itself, which is also achievable with standard compartmental models such as SIR or SEIR. The impact of different vaccination scenarios on infection dynamics has been explored through a set of illustrative counterfactual simulations. These scenarios compare only a few fixed cases (actual, no vaccination, delayed start, early stop) rather than systematically varying vaccination rates, and should be read as illustrative examples rather than a comprehensive scenario analysis. Furthermore, the comparison of optimisation approaches has highlighted the strengths and limitations of each method, offering a nuanced understanding of their applicability in different scenarios. Overall, these findings contribute to a better understanding of COVID-19 dynamics and provide a potentially useful framework for informing decision-making and intervention planning.

Several results support the effectiveness of the automated model discovery approach. First, the model discovered by SINDy already captures the main trends of the pandemic using the global coefficients, without any optimisation. After applying the optimisation methods, the model reaches an R² of 0.87 [0.83, 0.94] at one week and 0.73 [0.60, 0.89] at ten days (Table 5), showing that the discovered structure provides a basis for accurate short-term predictions within the model. As detailed in Results, these confidence intervals widen substantially at longer horizons, and performance differences between optimisation methods are not statistically distinguishable beyond approximately 7–10 days. Second, repeating the discovery process with different smoothing windows (Table 6) and different kernel parameters (Fig 14) consistently recovers the same set of active terms with the same signs, confirming that the result is not an artefact of a specific pre-processing choice. Third, the walk-forward validation (Fig 11) shows that the model performs consistently across most phases of the pandemic, though reliability decreases near the peak of a wave, when predictions become more sensitive to the model coefficients. Together, these results suggest that SINDy identified a meaningful mathematical representation of the infection dynamics. These results would be difficult to obtain with a fixed, low-dimensional classical SIR model without additional time-varying parameters, delay terms, or data-driven extensions.

Key to our findings was the development of a model that integrates newly derived features (infectiveness and antibody) through sophisticated data processing techniques (Fig 4). This integration has significantly enhanced the accuracy of our predictions, providing deeper insights into the temporal dynamics of the virus's transmission and the effectiveness of vaccination campaigns.

The exploration of various vaccination scenarios suggests that targeted immunisation strategies may play an important role in shaping the trajectory of the virus. These model-based scenarios illustrate how different policy interventions could alter predicted infection trajectories (Fig 8), thus offering exploratory insights into public health strategy and response planning. Importantly, they also highlight that such interventions require time before their effects on infection dynamics become visible.

Moreover, our comparative analysis of different optimisation approaches, local coefficient adjustment, time-dependent coefficient adjustment, and neural-augmented ODE adjustment, highlights the distinct advantages of each method in capturing the complex interplay of epidemiological data and external factors influencing pandemic trends (Fig 11, Table 5).

Despite their success in reflecting pandemic dynamics, all the models we applied are based on ODEs, which lack the hidden states used in methods like hidden Markov models or Kalman filters. While we incorporate historical context through the convolution of the infections and vaccinations, our approach primarily relies on data at individual time points and optimized coefficients from prior periods. An alternative to our convolution-based approach for handling temporal delays would be delay embedding methods, such as Takens’ embedding, which reconstruct the state space from lagged copies of the observed variables. However, this would substantially increase the number of state variables and, consequently, the size of the SINDy candidate library, making the sparse regression less stable and the discovered equations more difficult to interpret. In contrast, the convolution-based features used in this study condense the delay information into single, epidemiologically meaningful variables while incorporating domain knowledge from the literature on infectious periods and vaccine-induced antibody responses. This simplicity may limit predictive accuracy compared to models that explicitly handle latent states, such as exponential curve fitting or Kalman filters. However, by avoiding hidden states, our approach offers the advantages of easier interpretation and effective scenario analysis. This is advantageous, because every term in the discovered equations has a clear interpretation. This transparency makes it possible to understand why the model produces a certain prediction and to communicate the reasoning to non-technical decision makers. Models with hidden states, while potentially more accurate, do not offer this level of interpretability. For example, in [33], a fractional-order model has been proposed to capture memory effects in epidemic dynamics. While these methods introduce additional flexibility, they rely on predefined model structures. Our approach avoids this by letting the data determine the form of the equations. A limitation of the pre-processing step is that the averaging reduces possible sub-weekly variations in the data. However, since the dominant time scales of COVID-19 dynamics (including incubation periods, infectious duration, and the effects of interventions) are of the order of several days to weeks, the averaging preserves the features most relevant to the modelling objectives of our study. Furthermore, since the model is derived from observational data, some coefficients may reflect confounding effects. For example, the positive contribution of the antibody-infection interaction term in the infectiveness equation may partly capture the effect of concurrent policy relaxation during the vaccination rollout rather than a purely behavioural response. Similarly, the scenario analysis assumes that coefficients from one lockdown period generalise to lockdowns generally, which has not been tested. Together, these models reveal the ongoing challenge of accurately modelling such complex systems. Future research should focus on refining these models to better account for emerging virus variants and shifts in public behaviour. For example, integrating mobility data, public transport usage, or social media activity as additional control signals in the SINDy framework could help the model capture behavioural changes in real time, rather than relying solely on reported case numbers and vaccination records. Similarly, data-driven approaches for jointly estimating the kernel parameters alongside the model coefficients could reduce the reliance on manual selection in the pre-processing step. Moreover, interdisciplinary approaches incorporating the insight of behavioural science and economics could enhance the model's ability to simulate more realistic human responses to public health policies.

A further limitation concerns model reliability during peak infection periods. The sensitivity analysis (Fig 15) shows that predictions become markedly less stable when initiated near the peak of a wave: a ± 5% perturbation of the infection coefficient produces substantially larger divergence in predicted trajectories near the peak than during the growth or decline phases. This occurs because the infection coefficient's influence on the model dynamics is strongest during the transition between growth and decline, making the trajectory highly sensitive to small deviations at that point. In practice, this means predictions generated at or near a wave's peak should be treated with more caution than those generated during steadier growth or decline phases — which is precisely when accurate forecasts are most valuable for decisions such as hospital capacity planning or the timing of interventions.

An important limitation of this study is that the differential equations were discovered from a single regional dataset (Thuringia, Germany) covering a specific period of the pandemic (March 2020 to February 2022). The specific terms and coefficients in the discovered equations are likely to depend on local factors such as population demographics, healthcare capacity, dominant virus variants, and the timing and nature of public health interventions. Therefore, the discovered equations should not be expected to transfer directly to other regions or future pandemics without re-running the model discovery process on local data. However, the SINDy-based framework itself is transferable: given sufficiently detailed time-series data from another setting, the same pipeline can be applied to discover locally appropriate equations. Validating this transferability across diverse epidemiological contexts is an important direction for future work.

Our study contributes to the field of epidemiological modelling by exploring a data-driven approach to predicting disease spread, and presents a framework that may support the management of public health crises. For example, during a future outbreak, health authorities could, in principle, use this approach to quickly discover the governing dynamics from early case data, simulate the effect of different intervention strategies (such as varying vaccination rates or reintroducing contact restrictions), and update their predictions as new data becomes available. The scenario analysis capability (Fig 8) and the short-term forecasting accuracy (Table 5) suggest that this framework could inform operational decisions at regional level, although prospective validation on independent datasets would be needed before operational deployment. As we continue to face the challenges posed by COVID-19 and other infectious diseases, such data-driven and interpretable tools will be important for guiding public health responses.
